# A synthetic cell microreactor with two types of interacting dynamic DNA-based pores

**DOI:** 10.1038/s41557-026-02124-7

**Published:** 2026-05-15

**Authors:** Sisi Fan, Longjiang Ding, Benjamin Renz, Allen P. Liu, Thomas Speck, Hao Yan, Stephan Nussberger, Na Liu

**Affiliations:** 1 2nd Physics Institute, University of Stuttgart, Stuttgart, Germany; 2 Max Planck Institute for Solid State Research, Stuttgart, Germany; 3 Department of Mechanical Engineering, University of Michigan, Ann Arbor, MI USA; 4 Institute for Theoretical Physics IV, University of Stuttgart, Stuttgart, Germany; 5 Biodesign Center for Molecular Design and Biomimetics, Arizona State University, Tempe, AZ USA; 6 Department of Biophysics, Institute of Biomaterials and Biomolecular Systems, University of Stuttgart, Stuttgart, Germany

**Keywords:** DNA nanotechnology, DNA and RNA

## Abstract

Reconstructing cellular complexity is a central challenge in synthetic biology, with profound implications for understanding life and advancing bio-inspired nanotechnologies. A critical step towards this goal is replicating the dynamic interplay among membrane components and their functions. Here we demonstrate a double-necked synthetic cell microreactor (DCM) that incorporates two dynamic, DNA-based pores in the membrane of a giant unilamellar vesicle. The formation of the DCM leverages a signalling pathway mediated by giant unilamellar vesicle membrane dynamics to coordinate interactions between light-responsive small pores and self-arranged sealable large pores. This system enables sequential, on-demand delivery of molecular reactants with high spatiotemporal precision. Using DCMs, we demonstrate confined biochemical reactions, including a glucose oxidase–myoglobin cascade, cytoskeleton-mimetic actin polymerization and bundling, cell-free Spinach RNA transcription and the synthesis of three-dimensional DNA crystals that extend beyond natural systems. By coupling orchestrated multistep signalling with dynamic control of membrane permeability, the DCM establishes a versatile platform for emulating and expanding the functional complexity of natural cellular systems.

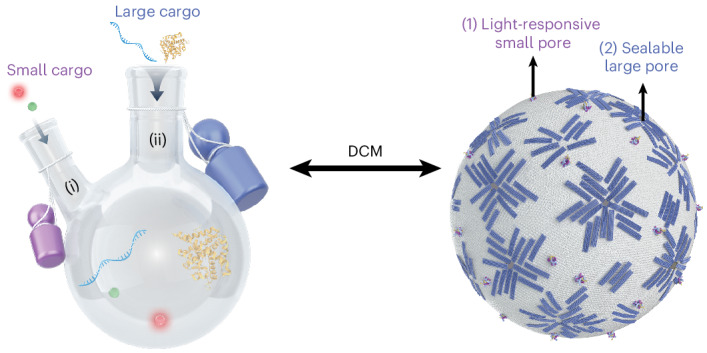

## Abstract (web summary)

Reconstructing coordinated membrane functions is a key challenge in synthetic cell engineering. Now it has been shown that a double-necked synthetic cell microreactor with two interacting, dynamic DNA-based pores can enable sequential, on-demand molecular transport with high spatiotemporal precision and support diverse confined biochemical reactions.

## Main

Cells function as highly efficient microreactors, orchestrating complex biochemical reactions within their membrane confines^1–3^. Membrane permeability plays a pivotal role in maintaining cellular function and adaptability across the biological spectrum, from bacteria to multicellular organisms^4–7^. This permeability is tightly regulated by specialized proteins that form membrane pores and channels, allowing cells to respond to external and intracellular cues. Despite differences in evolutionary origins and biological contexts, the formation of membrane-spanning structures is governed by specific signalling pathways that ensure precise timing and spatial localization. For instance, the mechanosensitive channel of large conductance in bacteria opens under elevated membrane tension, releasing ions and small solutes to prevent cell lysis^8^. In eukaryotic cells, Bcl-2-associated X (BAX) oligomerizes on the mitochondrial outer membrane in response to cellular distress, forming pores that release cytochrome *c* and activate apoptosis^9^. Although their biological consequences differ, cell survival in prokaryotes versus programmed death in eukaryotes, both mechanosensitive channel of large conductance and BAX exemplify how signalling pathways and intracellular orchestration precisely regulate membrane permeability to mediate critical life-or-death decisions. These examples underscore the shared regulatory principles that shape membrane transport across life forms.

Giant unilamellar vesicles (GUVs) have been widely adopted as model compartments for reconstituting biochemical processes under cell-like confinement^10,11^. A variety of biological pores and transporters, including protein pores^12^, ion channels^13^ and ionophores^14^, have been incorporated into lipid membranes to control molecular exchange. These systems have provided valuable insights into reaction dynamics and compartmentalized regulation. In recent years, DNA nanotechnology has opened new avenues for biomimetic engineering, notably through the development of DNA nanopores^15,16^. Unlike their protein-based counterparts, DNA nanopores offer a fully synthetic and highly programmable platform^17–26^. Their structural addressability allows precise control over pore geometry^27–33^, while their responsiveness to external stimuli, such as light^34^, nucleic acid strands^35–39^, temperature^40^, proteins^41^ or voltage^42^, enables reversible, on-demand regulation of membrane permeability. Furthermore, the intrinsic modularity of DNA-based assemblies facilitates the integration of multiple functionally distinct pores into a single membrane, paving the way for coordinated transport systems and signal-processing networks^43^. Despite these advances, most synthetic cells reported so far employ a single type of DNA pore with a predefined structure and function, primarily to mediate molecular transport across membranes. These pores typically operate in isolation, lacking mutual interaction or participation in membrane dynamics, and do not yield emergent cellular phenomena.

Here, we demonstrate a double-necked synthetic cell microreactor (DCM), comprising a GUV and two types of dynamic DNA-based pores, differing in size and function. The DCM enables sequential and precisely regulated transport of reactants for confined biochemical reactions with programmable permeability and spatiotemporal control. One neck consists of light-responsive small pores (SPs), while the other features self-arranged large pores (LPs). The indirect interaction between SPs and LPs during DCM formation is governed by a multistep signalling pathway mediated through the membrane dynamics of the GUV. The permeability of the DCM is synergistically modulated by SPs and LPs. While SPs allow the transport of ions and small molecules controlled by light, LPs enable the passage of macromolecules, such as single-stranded DNA (ssDNA), DNA motifs and proteins, regulated by DNA fuels. Equipped with these coordinated dynamic DNA-based pores, the DCM supports biochemical reactions by facilitating the on-demand delivery of diverse reactants. This capability is exemplified by the glucose oxidase (GOx)–myoglobin cascade, the reconstruction of actin polymerization and bundling and the cell-free Spinach RNA transcription, as well as the confined synthesis of three-dimensional (3D) DNA crystals. The DCM showcases the potential to replicate facets of the functional complexity in natural cellular systems by employing fully synthetic and programmable building blocks. Our system establishes a paradigm for programmable interpore signalling within synthetic cells. Unlike prior systems that focused on static pore behaviour or isolated gating events, the DCM reveals a causal relationship in which the state of one pore type regulates the formation and function of another. Coordination of pore activity through membrane dynamics gives rise to emergent, system-level behaviours, such as reversible LP formation and spatiotemporally regulated biochemical reactions. By bridging structural DNA nanotechnology and membrane biophysics, this work opens an avenue towards programmable, signal-driven processes in synthetic compartments.

## Results

### Signalling pathway during DCM formation

**Fig. 1:**
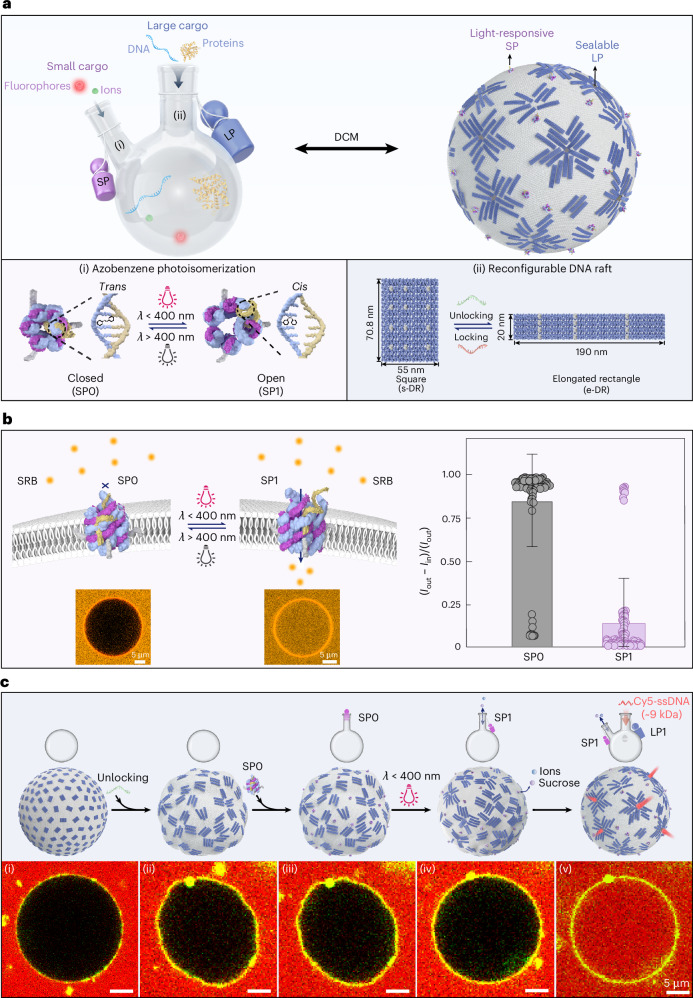
DCM. **a**, A schematic of the DCM, where two dynamic, DNA-based pores synergistically facilitate molecular transport across the GUV membrane with high spatiotemporal control. One neck is represented by light-responsive SPs. The SP featuring an azobenzene-modified lid strand switches between open and closed states via azobenzene photoisomerization under UV and visible light, respectively. The other neck is represented by sealable LPs, formed through the self-arrangement of reconfigurable DRs on the GUV membrane. **b**, SP transitions between its open (SP1) and closed (SP0) states under UV and visible light illumination, respectively, enabling the transport of small molecules, such as SRB across the GUV membrane. *(I*_out_ *−* *I*_in_)/*I*_out_ measurements of the SRB influx are shown for SP0 and SP1. Data represent the mean ± s.d. from three independent experiments. *n* = 110 in both cases. **c**, LP formation is induced via a signalling pathway identified using a Cy5-ssDNA (~9 kDa) influx assay. Introducing unlocking strands initiates the transition from s-DRs to e-DRs, leading to GUV deformations. Insertion of SP0 and its subsequent opening (SP1) by UV light triggers GUV shape recovery, leading to LP formation (LP1). Source data

The DCM consists of a GUV (1,2-dioleoyl-*sn*-glycero-3-phosphocholine (DOPC; 0.05 mol% Atto655-DOPE)) as the reactor container, with dynamic SPs and LPs serving as the two necks (Fig. 1a). The light-responsive SPs form the first neck. The SP is a barrel-shaped structure (~12.5 nm in height, ~2 nm inner diameter) with an azobenzene-modified lid strand, allowing reversible light-controlled opening and closing (Fig. 1a(i) and Supplementary Fig. 1). It permits diffusion of small molecules on a minute timescale, as previously characterized^34^. This lid can open and close the SP in response to light. The as-fabricated SPs are in the closed state. UV illumination (*λ* = 365 nm) switches the azobenzene from its *trans* to *cis* state, opening the pore, while visible light (*λ* = 450 nm) reverts the azobenzene to its *trans* state, closing the pore. Upon insertion into the GUV membrane via cholesterol anchors, SPs enable light-gated transport of small molecular cargo across the membrane. The confocal fluorescence images in Fig. 1b demonstrate that UV illumination triggers the opening of SPs, facilitating the influx of sulforhodamine B (SRB) into the GUV. The normalized fluorescence intensity difference *(I*_out_ *−* *I*_in_)/*I*_out_ (ref. ^44^), which quantifies the disparity between the fluorescence intensity inside the GUV (*I*_in_) and in the surrounding bulk solution (*I*_out_), reveals a sharp contrast between the open (SP1) and closed (SP0) states of the light-gated SPs (Fig. 1b). The few outliers observed in SRB influx arise from the intrinsic heterogeneity of GUVs and stochastic SP incorporation. However, the overall trend remains robust across large-population statistical analyses. A control experiment confirms that UV exposure does not affect the permeability of bare GUVs (Supplementary Fig. 2). In addition, statically open (sta-SP1) and closed (sta-SP0) pores, identical in structure but lacking the azobenzene modification, are assembled and examined in the influx experiment (Supplementary Fig. 3). The normalized fluorescence intensity differences of sta-SP1 and sta-SP0 are comparable to those of SP1 and SP0, respectively, confirming the effective gating performance of SPs regulated by light. The light-controlled closure of SPs is further verified by fluorescence recovery after photobleaching (FRAP). Following visible light (*λ* = 450 nm) illumination, photobleaching of SRB within the GUV leads to an immediate drop in fluorescence intensity. No fluorescence recovery over time is observed (Supplementary Fig. 4), demonstrating the switching reversibility of SPs. In addition to fluorophores, other small molecules essential for cellular activities, such as ions, can also be transported into GUVs via SPs on demand, as demonstrated in the Ca^2+^ influx experiment (Supplementary Fig. 5).

Recently, we have demonstrated that DNA origami rafts (DRs) can undergo reversible conformational changes on GUV membranes, switching from a square shape (s-DR, 70.8 nm × 55 nm, aspect ratio ~1.3) to an elongated rectangle shape (e-DR, 190 nm × 20 nm, aspect ratio ~9.5) upon the addition of DNA fuels^45^ (Fig. 1a(ii)). This self-arrangement of DRs from an isotropic to a short-range local order (Supplementary Fig. 6) generates steric pressure, inducing membrane bending. The localized deformation collectively translates into stress, driving large-scale morphological changes of the GUV^45^. Assisted by the statically open protein pore OmpF, the locally ordered DRs perforate the membrane during GUV shape recovery, forming large membrane pores. In this work, we extend the generality of this working mechanism by replacing protein pores with light-responsive SPs to induce DNA-based, sealable LPs mediated by GUV membrane dynamics. This enables the creation of the DCM entirely from synthetic building blocks, which can be independently controlled to facilitate programmable and dynamic coordination, thereby driving emergent complexity in synthetic reaction systems.

As shown in Fig. 1c(i) and Supplementary Fig. 7a, when only s-DRs (Atto488 labelled) are bound to the membrane, the GUV maintains a spherical shape. The addition of unlocking strands triggers the transition from s-DRs to e-DRs, resulting in prominent membrane deformations (Fig. 1c(ii) and Supplementary Fig. 8). When SPs in their closed state (SP0) are integrated into the GUV membrane, the deformations persist and no apparent influx of ssDNA (~9 kDa, Cy5 labelled) is observed (Fig. 1c(iii) and Supplementary Fig. 7b). Upon UV illumination, the opening of SPs (SP1) allows the exchange of small solutes (for example, sucrose, Na^+^, Mg^2+^ and Cl^−^) across the membrane, rapidly restoring osmotic balance. This process is further supported by the observed Ca^2+^ influx detected using the Ca^2+^-responsive fluorophore Fluo-8 (Supplementary Fig. 9). During this process, the GUV gradually returns to its spherical shape, yet no influx of ssDNA is detected (Fig. 1c(iv)). Only after the GUV fully recovers its spherical shape does the transport of ssDNA start, indicating the formation of LPs (LP1; Fig. 1c(v) and Supplementary Figs. 7c and 8) through interactions between the self-arranged e-DRs and the GUV membrane. The influx reaches equilibrium after ~30 min (Supplementary Fig. 10). These LPs continue to facilitate transmembrane transport of ssDNA even after 2 days (Supplementary Fig. 11), demonstrating their non-transient nature and long-term stability. Also, s-DR-bound GUVs integrated solely with light-responsive SPs in the open state (SP1) show no influx of ssDNA due to size exclusion (Supplementary Fig. 12). e-DR-bound GUVs with light-responsive SPs in the closed state (SP0) exhibit neither shape recovery nor ssDNA influx (Supplementary Fig. 13). Together with a theoretical framework that qualitatively elucidates the underlying mechanism (Supplementary Fig. 14 and the discussion immediately following), these experiments demonstrate that the LP formation is not random but is tightly regulated by a defined signalling pathway. This pathway during DCM formation involves the input of unlocking strands to trigger the transition from s-DRs to e-DRs, GUV deformations, the opening of SPs (SP1) by UV light, recovery of the GUV’s shape and eventually, large pore formation (LP1)^45^. Thus, the indirect interaction between SPs and LPs (Supplementary Fig. 15), mediated by the membrane dynamics of the GUV, leads to the creation of the DCM equipped with two fully synthetic, dynamic necks for orchestrated molecular delivery.

### Dynamic control over the DCM permeability

**Fig. 2:**
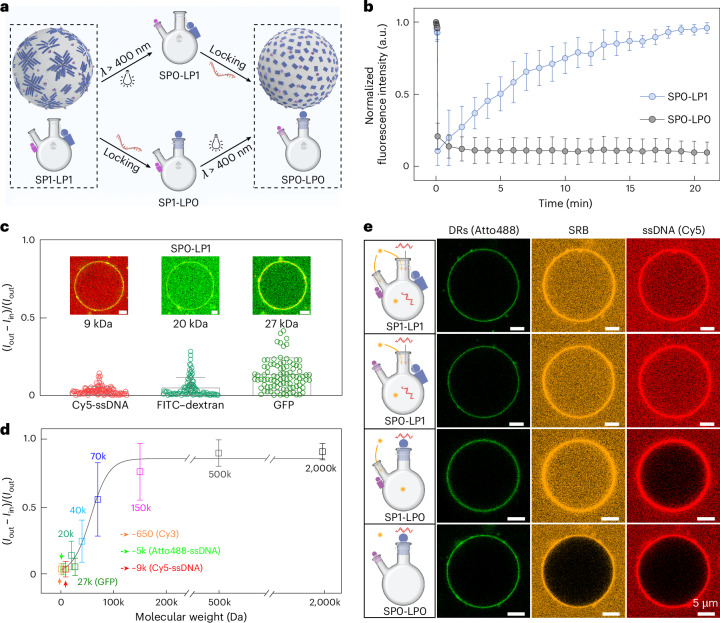
Dynamic control over the DCM permeability. **a**, The on/off configurations of SPs and LPs establish four distinct states of the DCM: SP1-LP1, SP0-LP1, SP1-LP0 and SP0-LP0, where ‘1’ and ‘0’ denote on and off states, respectively. **b**, The sealing efficiency of LPs. At SP0-LP0, photobleaching of ssDNA-Cy5 within the GUV causes a sharp fluorescence intensity decrease without recovery (grey), confirming successful LP closure. Conversely, at SP0-LP1, fluorescence recovers rapidly after photobleaching (blue). Data are presented as mean ± s.d. (*n* = 3 for both cases). **c**, The efficient transport of large cargo, including ssDNA (9 kDa), dextran (20 kDa) and GFP (27 kDa), is demonstrated via influx assays, measured as (*I*_out_ *−* *I*_in_)/*I*_out_. Inset: scale bars, 5 μm. Data represent mean ± s.d. from three independent experiments. *n* = 97, 88 and 96 for ssDNA, dextran and GFP, respectively. **d**, The dimension estimation of LPs based on influx assays using molecules of varying molecular weights. Data represent mean ± s.d. from three independent experiments. *n* = 114, 114, 77, 112, 110, 114, 114, 117, 120 and 114, for Cy3, 5 kDa ssDNA, 9 kDa ssDNA, 20 kDa dextran, GFP, 40 kDa dextran, 70 kDa dextran, 150 kDa dextran, 500 kDa dextran and 2,000 kDa dextran, respectively. **e**, The dynamic interplay between SPs and LPs allows precise temporal control over DCM permeability, facilitating orchestrated cargo transport. Source data

The dynamic interplay between SPs and LPs enables the DCM to adopt four distinct states based on their on/off configurations (Fig. 2a). Visible light illumination closes SPs, transitioning the DCM from SP1-LP1 to SP0-LP1. Subsequent addition of locking strands reconfigures e-DRs back to square DNA rafts (R-s-DRs), sealing LPs and resulting in SP0-LP0. Alternatively, the DCM transitions from SP1-LP1 to SP0-LP0 by first adding locking strands to reach SP1-LP0, followed by visible light illumination. To evaluate the sealing efficiency of LPs, FRAP experiments are conducted at SP0-LP0 and SP0-LP1, respectively, for direct comparison. At SP0-LP0, photobleaching of Cy5-ssDNA within the GUV results in an immediate decrease in fluorescence intensity without recovery over time (Fig. 2b, grey curve, and Supplementary Fig. 16a), confirming the successful closure of LPs. By contrast, at SP0-LP1, fluorescence recovers rapidly after photobleaching (Fig. 2b, blue curve, and Supplementary Fig. 16b). Beyond ssDNA, other large molecular cargo, such as fluorescein isothiocyanate (FITC)–dextran molecules (∼20 kDa) and green fluorescent protein (GFP, ∼27 kDa) can also be efficiently transported through LPs (Fig. 2c and Supplementary Fig. 17). In addition, the dimension of LPs can be determined through influx assays using molecules of varying molecular weights^44^ (Fig. 2d and Supplementary Figs. 18 and 19). The population distribution centres at 0.56 for 70 kDa FITC–dextran, suggesting that the dimension of LPs is ~15 nm based on the hydrodynamic diameter of the dextran molecule^46^. Consistently, the DCMs also enable size-selective efflux of encapsulated molecules, demonstrating bidirectional and regulated transport (Supplementary Fig. 20).

A key feature of the DCM is its dynamically controllable permeability through the coordination of SPs and LPs. To demonstrate this functionality, influx assays are performed with Cy5-ssDNA and SRB coexisting in the exterior of GUVs (Fig. 2e and Supplementary Fig. 21), while e-DRs are labelled with Atto488 to define membrane boundaries. When the DCM is at SP1-LP1, Cy5-ssDNA is transported via LPs, while SRB diffuses through both LPs and SPs. At SP0-LP1, where SPs are closed upon visible light exposure and LPs remain open, both Cy5-ssDNA and SRB are transported into the GUV lumen through LPs. At SP1-LP0, where SPs are opened by UV illumination and LPs are sealed by reconfiguration from e-DRs to R-s-DRs, only SRB influx occurs because size exclusion prevents Cy5-ssDNA entry. Finally, at SP0-LP0, no influx is observed. In addition, a detachable raft strategy is developed to enable reliable system resetting and LP re-opening through toehold-mediated raft detachment, membrane resealing and reconfiguration upon resupply of s-DRs (Supplementary Figs. 22 and 23). Furthermore, the DCM platform exhibits stability and functional compatibility under physiologically relevant conditions^47,48^ (Supplementary Fig. 24).

In typical GUV-confined reactions, large reactants are often pre-encapsulated during GUV formation or mechanically injected. Here, the coordination between SPs and LPs enables sequential, on-demand transport of different reactants into DCMs, as exemplified by the implementation of an enzyme cascade. Enzyme cascades are a hallmark of biochemical organization in living systems, where sequential reactions are orchestrated with spatial and temporal precision. A GOx–myoglobin cascade is established within the DCM, regulated through the stepwise transport of distinct reactants (Supplementary Fig. 25). The realization of this cascade within a membrane-confined environment highlights that the DCM can go beyond single-step conversions to achieve programmable, pathway-like biochemical processing. In the following sections, we further showcase how these unique features facilitate biochemical reactions inside DCMs with programmable transport and precise spatiotemporal control.

### Actin polymerization and bundling within the DCM

Actin polymerization and bundling are vital biological processes that contribute to cell shape, motility and intracellular transport^49,50^. G-actin, a globular protein, polymerizes into long, filamentous structures (F-actin) within cells^51,52^. These filaments are then organized into bundles by crosslinking proteins such as fascin^53–55^, which enhances their structural stability and mechanical strength (Supplementary Fig. 26).

**Fig. 3:**
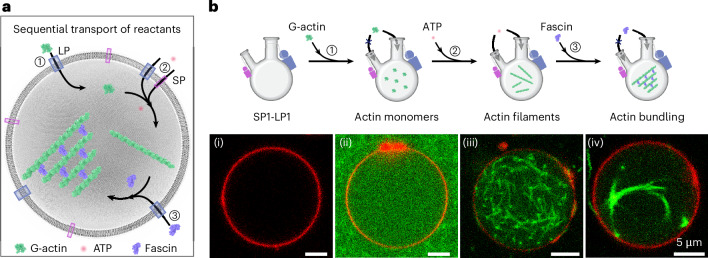
Actin polymerization and bundling within the DCM. **a**, A schematic of sequential transport of reactants, enabling actin polymerization and bundling within the DCM. **b**, Intermediate steps and corresponding confocal fluorescence images of the DCM: (i) DCM is initially at SP1-LP1; (ii) after G-actin (~42 kDa) transport through LPs, the lumen exhibits homogeneous fluorescence; (iii) ATP is delivered via both SPs and LPs, binding to G-actin to initiate polymerization; and (iv) fascin (~55 kDa) is introduced into the DCM through LPs, crosslinking the polymerized actin filaments. Lipids (Atto655), filament staining and visualization (Atto488-phalloidin).

The DCM provides a controlled, cell-sized environment to reconstruct actin networks through on-demand delivery of reactants (Fig. 3a). DRs and G-actin are labelled with Atto488 and rhodamine, respectively. During the transition from s-DRs to e-DRs, GUV deformation occurs, followed by SP activation with UV light and subsequent GUV shape recovery. This signalling pathway enables the formation of LPs, facilitating the influx of G-actin (~42 kDa) and its uniform distribution within the lumen (Supplementary Fig. 27). After verifying G-actin transport, unlabelled G-actin is used in actin polymerization and bundling experiments with Acti-stain 488 phalloidin employed for filament staining and visualization (Fig. 3b). Initially, the DCM lumen appears dark at SP1-LP1 (Fig. 3b(i)). G-actin is then transported through LPs, while Acti-stain 488 phalloidin is added. The lumen exhibits homogeneous fluorescence (Fig. 3b(ii)). ATP is subsequently delivered via both SPs and LPs, binding to G-actin to initiate polymerization. The formation of elongated, filamentous structures confirms F-actin assembly (Fig. 3b(iii) and Supplementary Fig. 28). Finally, fascin (~55 kDa) is introduced into the DCM through LPs, crosslinking the polymerized actin filaments. This crosslinking creates densely packed actin bundles and interconnected networks, emulating the organizational complexity of cellular actin cytoskeleton (Fig. 3b(iv) and Supplementary Fig. 29).

### Cell-free RNA transcription within the DCM

**Fig. 4:**
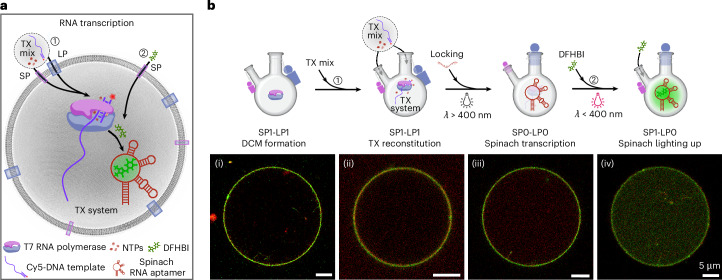
Spatiotemporally controlled cell-free RNA transcription within the DCM. **a**, A schematic of Spinach RNA aptamer synthesis and fluorescence visualization in a cell-free transcription (TX) system regulated by spatiotemporally regulated transport of reactants. **b**, The stepwise workflow and corresponding confocal fluorescence images: (i and ii) DNA templates and TX mix are introduced into DCMs pre-encapsulating T7 RNA polymerase; (iii) both LPs and SPs are closed to create a confined environment and prevent leakage after removal of exterior templates; and (iv) SPs are subsequently re-opened to allow DFHBI influx, enabling fluorescence visualization of RNA production. Green, DRs and Spinach–DFHBI complex; red, lipids and Cy5-labelled DNA template.

RNA transcription is a fundamental process of cellular gene expression and regulation^56–58^. To demonstrate the versatility of our platform, we next implement cell-free RNA transcription within the DCM (Fig. 4a). This involves the sequential delivery of a transcription mixture (TX mix) and a fluorescence indicator, recapitulating key aspects of transcriptional regulation.

As illustrated in Fig. 4b, the Spinach RNA aptamer is employed as a model reporter, emitting fluorescence upon binding to the fluorogen 3,5-difluoro-4-hydroxybenzylidene imidazolinone (DFHBI; ~252 Da), thus providing a convenient optical readout^58,59^. T7 RNA polymerase (~99 kDa) is pre-encapsulated in GUVs during preparation and remain confined after DCM formation in the SP1-LP1 state. The TX mix, containing Cy5-labelled DNA templates (~35 kDa), NTPs and transcription buffer, is introduced externally and transported into the DCM. Both LPs and SPs are subsequently closed to create a sealed, cell-like compartment and prevent leakage after removal of residual exterior reagents. Following incubation at 37 °C for 2 h, SPs are re-opened by UV illumination to allow DFHBI influx. Clear green fluorescence from the Spinach–DFHBI complex is observed within the DCM lumen, confirming RNA production. These results demonstrate that the DCM can achieve controlled, cell-free gene expression within a confined, programmable synthetic compartment. Furthermore, large field-of-view confocal fluorescence images (Supplementary Fig. 30) illustrate the high functional fidelity and reliability of our system.

### Confined synthesis of 3D DNA crystals within the DCM

Hierarchical DNA self-assembly, where small DNA motifs form larger structures through multistep processes, substantially expands the complexity and diversity of DNA-based materials^17^. In 2009, Seeman and Mao introduced the rational design of a 3D DNA crystal based on the tensegrity triangle, creating a well-ordered macromolecular 3D crystalline lattice^60^. Later, they improved sticky-end cohesion via 5′-phosphorylation, effectively promoting DNA crystal nucleation and growth^61–63^.

**Fig. 5:**
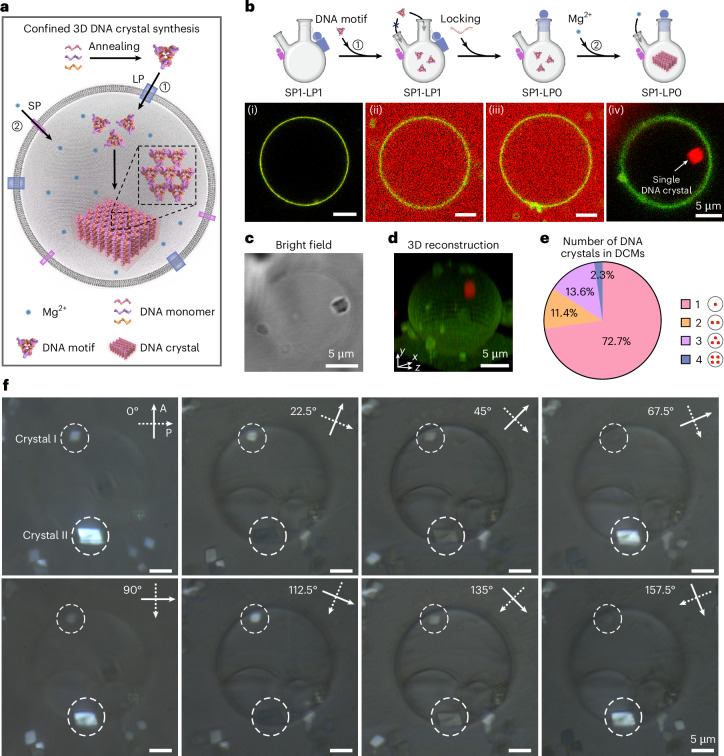
Confined synthesis of 3D DNA crystals within the DCM. **a**, A schematic of 3D DNA crystallization within cell-sized confinements. **b**, Intermediate steps and corresponding confocal fluorescence images of the DCM: (i) DCM is initially at SP1-LP1; (ii) DNA triangle motifs are introduced through LPs into the DCM; (iii) LPs are sealed by adding locking strands; and (iv) Mg^2+^ is incrementally supplemented into the DCM through SPs to induce DNA crystallization. Green, DRs; red, Cy5-labelled DNA motifs. **c**,**d**, Bright-field image (**c**) and *z*-stack 3D visualization (**d**) of a DCM containing a single DNA crystal. **e**, Statistical analysis of the number of DNA crystals formed in individual DCMs. *n* = 44. **f**, Birefringent properties of the DNA crystals under crossed polarized light. The alternating bright and dark appearances of the two DNA crystals under rotating polarizers confirm the presence of distinct crystalline axes. The orientations of the polarizer (P, dashed arrow) and analyser (A, solid arrow) are indicated by arrows. Source data

Traditionally, DNA crystals are synthesized in bulk solution drops, requiring prolonged evaporation or slow annealing^60–66^. However, the confined growth of DNA crystals remains largely unexplored, despite its promising scientific and technical potential. For instance, integrating DNA crystals into synthetic cells could advance their functional capabilities by providing scaffolds to spatially organize biomolecules and enzymes with high precision. We demonstrate the synthesis of 3D DNA crystals within cell-sized confinements. As a model system, we use Seeman and Mao’s 3D rhombohedral DNA crystals (Fig. 5a), where DNA triangle motifs connect via sticky-end cohesion modified by 5′-phosphorylation^63^. This crystallization process is highly sensitive to assembly conditions, requiring precise control over DNA triangle motif concentration and buffer parameters, such as ionic strength and sucrose concentration. Optimized buffer conditions of 50 mM sucrose (Supplementary Fig. 31) and 10 mM Mg^2+^ (Supplementary Fig. 32) are first identified for crystal growth at DNA triangle motif concentrations exceeding 10 µM (Supplementary Fig. 33). A lower Mg^2+^ concentration of 5 mM proves insufficient to induce crystallization.

To synthesize DNA crystals, DCMs are prepared with both SP and LPs open (SP1-LP1; Fig. 5b(i)). DNA triangle motifs, pre-assembled by annealing three composing DNA strands, are introduced into the DCMs through LPs in the presence of 5 mM Mg^2+^. Cy5-labelled DNA triangle motifs distribute homogeneously within the DCM lumen (Fig. 5b(ii)). LPs are then sealed by adding locking strands, converting e-DRs to R-s-DRs (Fig. 5b(iii)). This sealing step confines the DNA motifs within the DCM, preventing efflux during subsequent steps. Mg^2+^ is incrementally supplemented into the DCM through SPs to achieve a final concentration of 10 mM. Stepwise Mg^2+^ supplementation is crucial because directly introducing 10 mM Mg^2+^ at the start would cause crystallization primarily outside the DCM, where reactants are abundant. Controlled Mg^2+^ delivery through SPs, combined with sealed LPs, ensures effective nucleation and crystal growth, as evidenced by the single-crystal formation within the DCM (Fig. 5b(iv)). The DNA crystals are formed inside the DCMs following a 2-day incubation at 22 °C. Figure 5c,d shows the bright-field and 3D reconstruction (Supplementary Fig. 34) images of a DCM containing a single DNA crystal with defined facets, characteristic of its crystalline nature.

DNA crystallization within DCMs demonstrates notable confinement effects. Confinement predominantly promotes nucleation at a single site (72.7%, *n* = 44; Fig. 5e), suppressing multinucleation events. Once nucleation initiates, it tends to dominate the growth process, outcompeting other potential sites owing to the depletion of DNA motifs in the confined space. As reactants are sequentially delivered into the DCM, the first site where Mg^2+^ reaches a critical nucleation concentration starts crystal growth, reducing supersaturation to levels insufficient for additional nucleation events. Furthermore, crystallization preferentially occurs near the DCM’s inner surfaces (Supplementary Fig. 35), probably because these surfaces provide favourable nucleation sites. As Mg^2+^ is introduced through SPs in the membrane, its concentration is probably higher near the inner surface, driving crystallization in that region. In addition, the confined volume of the DCM limits crystal growth, favouring boundary-aligned development. The crystalline nature of the 3D DNA crystals is validated through birefringence analysis using crossed polarizers. In a DCM containing two DNA crystals (Fig. 5f), the alternating bright and dark appearances under rotating polarizers confirm the presence of distinct crystalline axes (also see Supplementary Fig. 36).

## Discussion

The DCM exemplifies a platform in synthetic cells, where orchestrated signalling and dynamic pore coordination emulate key features in natural cellular processes (Supplementary Table 1). Through the indirect interaction between SPs and LPs, the DCM offers high spatiotemporal control over molecular transport and confined biochemical reactions. We have demonstrated this capability across multiple model processes, including a GOx–myoglobin enzyme cascade to achieve pathway-like reactions, actin polymerization and bundling to mimic cytoskeletal organization, and cell-free Spinach RNA transcription for controlled gene expression. In addition, the synthesis of 3D DNA crystals within the confined environment of the DCM underscores its potential towards structural organization at subcellular scales, demonstrating capabilities that extend beyond natural systems.

From a futuristic perspective, the DCM lays a foundation for addressing critical challenges in synthetic biology and materials science^67,68^. Expanding the repertoire of stimuli-responsive DNA pores could enable the integration of multiple signalling pathways, creating synthetic cells with enhanced complexity and adaptability. Leveraging the DCM for multiscale molecular assembly could lead to hierarchical materials, where DNA crystals or polymerized networks serve as scaffolds for higher-order organization. Moreover, exploring interactions between DCMs and external environments could unlock opportunities in cellular communication and hybrid systems. For example, coupling DCMs with engineered protein pores or membrane-associated enzymes might yield hybrid platforms that combine the precision of DNA nanotechnology with the biochemical versatility of proteins. Such systems could serve as models for studying membrane dynamics, as well as prototypes for next-generation therapeutic delivery systems or artificial organelles. Moreover, DCMs could also be useful for high-throughput, cell-free synthesis of proteins and antibodies in the future.

While the DCM enables programmable control of transport through the dynamic coordination of DNA pores, its intrinsic molecular selectivity remains moderate as both SPs and LPs allow passage of a range of small molecules. The present selectivity is therefore primarily state dependent, governed by the reversible opening, closing and formation of pores rather than by fixed steric or electrostatic exclusion. Future designs may improve molecular specificity by incorporating sequence-controlled gating motifs or chemically functionalized channels that discriminate molecules based on charge, size or affinity, thereby expanding the precision of transport regulation in DNA-based microreactors.

In conclusion, the DCM marks a step towards reconstituting the complexity and functionality of natural cells with fully synthetic building blocks. Its dynamic, sequential and precise control over molecular transport opens avenues for innovation in synthetic biology and materials science. Future developments may enable synthetic systems and biologically inspired microfactories to not only mimic the capabilities of natural cells, but also achieve unprecedented control over biochemical processes for applications in targeted drug delivery, programmable chemical synthesis and beyond^69^.

## Methods

### Assembly and characterization of the SPs

All DNA sequences of the SPs are provided in Supplementary Table 2. The SPs were assembled as follows. Equimolar mixtures of DNA oligonucleotides (10 μM each) in a 1× TE buffer, supplemented with 16 mM MgCl_2_ were annealed for ~3 h by the following thermal annealing protocol: 95 °C for 10 min, from 85 °C to 20 °C at a rate of 2 min °C^−1^. The folded SPs were analysed with 10% native polyacrylamide gels. The electrophoresis was conducted in Tris–borate–EDTA buffer with 16 mM MgCl_2_ at a constant voltage of 200 V for 40 min. The gels were scanned by a UV transilluminator after staining with SYBR Gold. The light intensity used to trigger SP opening in the GUV experiments was the same to that used for SPs in bulk (Supplementary Fig. 1). Specifically, UV illumination at 365 nm (5.6 kJ m^−2^) and visible illumination at 450 nm (14.4 kJ m^−2^) were applied.

### Assembly and purification of the s-DRs

The s-DR was designed using Cadnano, and the detailed sequences are provided in Supplementary Table 3. Assembly of the s-DRs was performed as follows: a long scaffold strand (7,560-nt circular single-stranded M13mp18 DNA, 20 nM) was folded into a square shape through interactions with 154 staple strands (100 nM each; Supplementary Table 4) in a 1× TE buffer supplemented with 1 mM EDTA and 12 mM MgCl_2_. The samples underwent a 10-h thermal annealing process using the following protocol: 95 °C for 5 min, then cooling from 85 °C to 24 °C at a rate of 10 min °C^−1^. For microscopy experiments, the s-DRs were purified via polyethylene glycol (PEG) precipitation. The folded s-DR sample was mixed at a 1:1 ratio with the PEG buffer (15% PEG 8,000 (w/v), 5 mM Tris, 1 mM EDTA and 505 mM NaCl). The mixture was centrifuged at 10,000*g* for 15 min at room temperature. The supernatant was carefully removed using a pipette and the remaining pellet was then dissolved in the imaging buffer (92.5 mM NaCl, 5 mM MgCl_2_ and 20 mM HEPES, pH 7.2, ~220 mOsm kg^−1^).

### Reversible conformation changes of DNA rafts on SLBs

SLBs were formed at 60 °C by the fusion of small unilamellar vesicles (SUVs) deposited in the imaging buffer (92.5 mM NaCl, 5 mM MgCl_2_ and 20 mM HEPES, pH 7.2) onto freshly cleaved mica. The SUVs were prepared as follows: DOPC (Avanti Polar Lipids) was dissolved in chloroform at a concentration of 25 mg ml^−1^ for SLB formation. A 40-µl aliquot of the DOPC stock solution was transferred into a clean glass vial, dried under a stream of nitrogen for 10 min and left overnight under vacuum to remove residual organic solvent. The dried lipids were rehydrated in 1 ml of double-deionized water and sonicated for 6 min using a Branson Ultrasonics Sonifier (60% duty cycle) to produce SUVs at a lipid concentration of 1 mg ml^−1^. After a 30-min incubation period, the mica surface was gently washed with 400 µl of imaging buffer to remove excess lipids and the SLB was slowly cooled to room temperature to minimize membrane defects. The purified s-DRs were incubated with the SLB for 40 min, allowing the cholesterol anchors to insert into the bilayer, thereby attaching the s-DRs to the membrane. Unbound s-DRs were removed by gently washing the sample twice with 400 µl of imaging buffer. The transformation of s-DRs into e-DRs was triggered by adding unlocking strands (Supplementary Table 5) at 40 °C after 1 h of incubation, with the temperature maintained using a heating plate. This dynamic transformation of s-DRs to e-DRs occurred on the membrane. Subsequently, the addition of locking strands (Supplementary Table 6) at 40 °C, followed by another 1-h incubation, reversibly reconfigured the e-DRs into R-s-DRs. For confocal imaging, the DNA rafts were labelled with Atto488 (Supplementary Table 7). For atomic force microscopy (AFM) imaging on the SLB interface, a Multimode VIII AFM with a Nanoscope V controller was used, equipped with an SNL-10 microcantilever tip (Bruker).

### Preparation of GUVs

GUVs were typically prepared using the Vesicle Prep Pro device (Nanion Technologies). First, 20 µl of lipid stock solution containing DOPC and Atto655-DOPE (99.95:0.05 molar ratio, 2.0 mg ml^−1^ in chloroform) was deposited onto ITO-coated slides, dried for 20 min and assembled into an electroformation chamber. Hydration was performed with 200 µl of 220-mM sucrose supplemented with desired molecules (for example, Fluo-8 or GOx), followed by the application of an alternating current electric field (3 V, 5 Hz) at 37 °C for 2 h. For GUVs encapsulating T7 RNA polymerase from the HiScribe T7 High Yield RNA Synthesis kit, emulsion transfer was used owing to the ionic content of the storage buffer. A 450-µl lipid-in-oil solution (3 mg ml^−1^) was prepared by vortexing and sonication, layered over 200 µl of imaging buffer in a 2-ml tube, and left undisturbed for 30 min before mixing with a 7-µl interior solution containing 220 mM sucrose and T7 RNA polymerase. Following centrifugation (4,500*g*, 10 min, 5 °C), GUVs were gently resuspended in the aqueous phase for subsequent experiments.

### Signalling-guided formation of the DCM

s-DRs (1.1 nM) with cholesterol modifications were mixed with GUVs at a s-DR:lipid molar ratio of 1:1,000 and incubated for 1 h to allow membrane binding. Unlocking strands were then added to induce the conformational transition from s-DRs to e-DRs, followed by a 1-h incubation. This process led to GUV deformation. Next, as-fabricated SP0 was introduced at a final concentration of 100 nM. After a 1-h incubation, a 1-min illumination at *λ* = 365 nm triggered the transition of SP0 to the open-state SP1, initiating GUV shape recovery and eventually leading to LP formation. This multistep signalling process resulted in the creation of the DCM. To confirm LP formation, an influx assay was performed using Cy5-ssDNA (~9 kDa) at a concentration of 1 µM. To revert SP1 to SP0, a 1-min illumination at *λ* = 450 nm was applied. To seal the LPs, locking strands were introduced to reconfigure e-DRs into R-s-DRs. All steps were conducted under isosmotic conditions.

### Laser-scanning confocal fluorescence microscopy

To minimize non-specific adhesion and protect GUVs during flowcell experiments, the 200-µl chambers (Ibidi) were coated with bovine serum albumin (BSA) before use by applying 100 μl of 2.0 mg ml^−1^ BSA solution to fully cover the channel surface, followed by complete removal of the solution. Confocal imaging was performed using an LSM 980 (Zeiss) laser-scanning microscope equipped with a C-Apochromat 60×/1.2 W oil immersion objective (Zeiss). For FRAP experiments, the equatorial plane of the GUV was selected. Further image analysis was conducted using a MATLAB program.

### GOx–myoglobin cascade

GOx (0.05 mg ml^−1^) was encapsulated in GUVs by electroformation. After DCM formation in the SP1-LP1 state, Amplex Red (5 μM) was added and allowed to diffuse across the membrane, followed by transport of Cy5-labelled myoglobin (2 μM) into the GUVs. Locking strands were then introduced, which transformed e-DRs to s-DRs and sealed LPs at 40 °C for 2 h. The samples were gently washed to remove external myoglobin. Glucose (1 mM) was subsequently added and transported through SPs (SP1) to initiate the cascade. For experiments with closed SPs (SP0), UV light exposure was applied before glucose addition.

### Actin polymerization and bundling

Unlabelled and rhodamine-labelled G-actin from rabbit skeletal muscle were purchased from Hypermol. To demonstrate its transport into the DCMs, rhodamine-labelled G-actin was used, while unlabelled G-actin was employed for actin polymerization and bundling reactions within the DCM. To maintain actin activity, a buffer containing 5 mM Tris–HCl, 0.2 mM CaCl_2_, 0.2 mM ATP, 1 mM DTT, 100 mM KCl and 2 mM MgCl_2_ at pH 8.0 was used in the experiment. G-actin (1 μM) was first transported into the DCMs via LPs. Once equilibrium was reached, ATP (1 mM) was introduced, diffusing into the DCMs through both pore types to trigger actin polymerization. The sample was then thoroughly washed with the buffer to remove actin outside the DCMs. Finally, fascin (~55 kDa, 0.5 μM) was added and transported into the DCMs via LPs to induce actin filament bundling.

### Cell-free Spinach RNA aptamer transcription

All reagents were used at the concentrations specified in the HiScribe T7 High Yield RNA Synthesis kit (New England Biolabs). After DCM formation in the SP1-LP1 state with GUVs encapsulating T7 RNA polymerase, TX mix containing reaction buffer, NTPs, DTT and Cy5-labelled DNA template^58^ (100 nM; Supplementary Table 8) was introduced and allowed to transport into the DCMs. Locking strands were then added to seal the LPs at 40 °C for 2 h, followed by UV illumination to close the SPs. After washing to remove external reactants, the samples were incubated at 37 °C for 2 h to allow RNA transcription. Finally, illumination at *λ* = 450 nm re-opened the SPs, permitting DHFBI (5 μM) to enter the DCMs and form fluorescent complexes with Spinach RNA.

### Three-dimensional DNA crystallization

DNA motifs (25 µM) were assembled by mixing the corresponding strands (L, S, and M in Supplementary Table 8) in TAE/Mg^2+^ buffer (5 mM) and gradually cooling from 95 °C to 40 °C over 1 h. The assembled DNA motifs were then introduced into the flow chamber, allowing transport into the DCMs through LPs at 40 °C for 2 h. Locking DNA strands were subsequently added to seal the LPs, maintaining the temperature at 40 °C for an additional 2 h. To achieve a final Mg^2+^ concentration of 10 mM, 0.5 µl of 500 mM MgCl_2_ was added, enabling Mg^2+^ transport into the DCMs through SPs. The sample was then incubated at 22 °C for 2 days.

## Online content

Any methods, additional references, Nature Portfolio reporting summaries, source data, extended data, supplementary information, acknowledgements, peer review information; details of author contributions and competing interests; and statements of data and code availability are available at 10.1038/s41557-026-02124-7.

## Supplementary information

Supplementary Information Supplementary Figs. 1–36, Tables 1–8 and References.

## Source data

Source Data Fig. 1 Statistical source data.

Source Data Fig. 2 Statistical source data.

Source Data Fig. 5 Statistical source data.

## Data Availability

All the data supporting the results of this study are included in this Article and its Supplementary Information. Where feasible, original datasets are included. In some cases, a single dataset comprises several hundred raw data files and is represented by processed data in the figures and Supplementary Information. Owing to the large volume of raw data and image files generated in this study, they are available from the corresponding author upon reasonable request. Source data are provided with this paper.
