# Supplementary material for: A synthetic cell microreactor with two types of interacting dynamic DNA-based pores
Source: Nat Chem. 2026 May 15;18(7):1325–33. doi: 10.1038/s41557-026-02124-7 (PMC13322974; doi:10.1038/s41557-026-02124-7)
Supplement: Supplementary file 1 — Supplementary Figs. 1–36, Tables 1–8 and References. [file 41557_2026_2124_MOESM1_ESM.pdf]

nature chemistry

Supplementary information https://doi.org/10.1038/s41557-026-02124-7

A synthetic cell microreactor with two types of interacting dynamic DNA-based pores

In the format provided by the authors and unedited

Table of contents

Supplementary Figures……..…………………………………...…………..…… 2

Supplementary Tables………………………………………………….……......32

References……………………………………………………………………...38

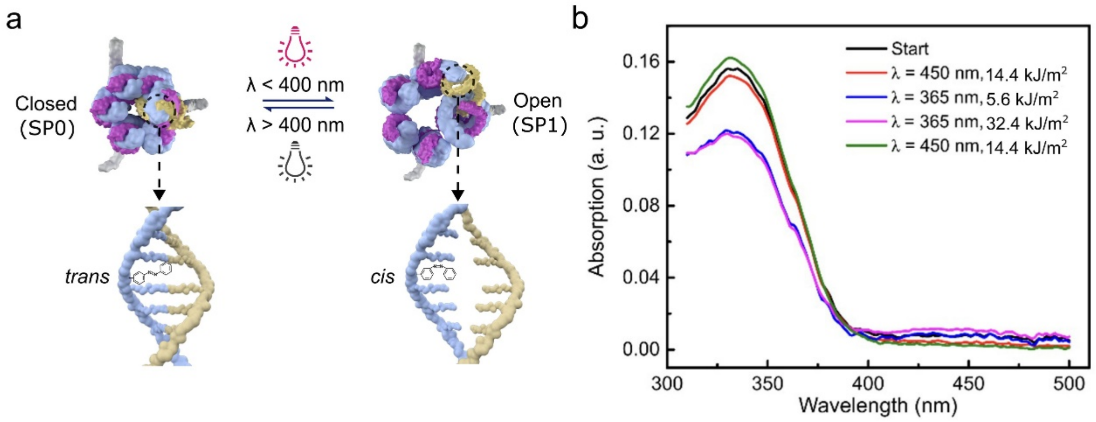

Figure S1. (a) Schematic illustration of azobenzene photoisomerization controlling the opening and closing of SPs. (b) Reversibility of azobenzene isomerization in SPs, demonstrated by UV-Vis spectra. The SPs fabricated under ambient light are in the closed state (SP0), as shown by the black curve. Illumination at $\lambda = 450$ nm (14.4 kJ/m^2) does not significantly alter the spectrum (red curve). When illuminated at $\lambda = 365$ nm (5.6 kJ/m^2), azobenzene isomerization from *trans* to *cis* is triggered, resulting in a decrease in intensity at the characteristic absorption wavelength of 340 nm (blue curve). Further exposure using a higher dose of UV light (32.4 kJ/m^2) does not notably affect the intensity (pink curve). Subsequent illumination at $\lambda = 450$ nm (14.4 kJ/m^2) nearly restores the original intensity (green curve).

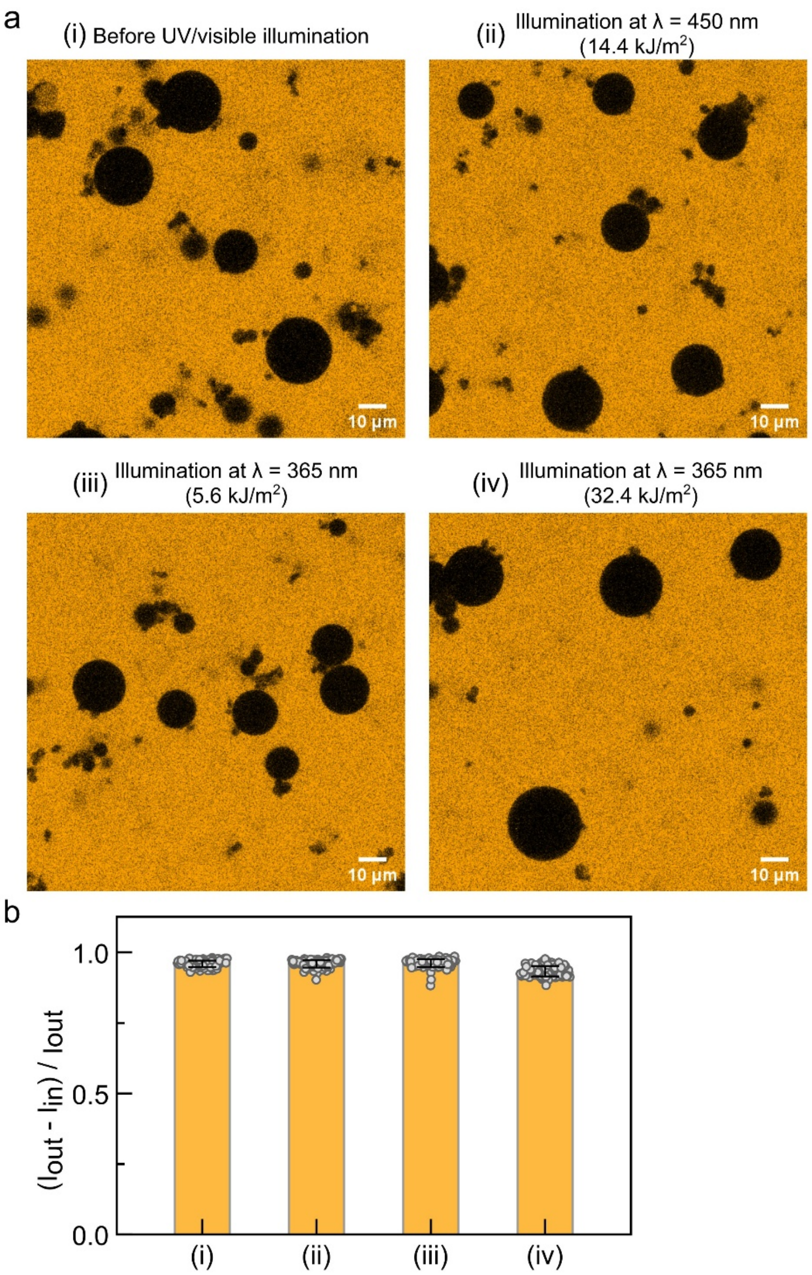

Figure S2. GUV membrane stability under different illumination conditions. (a) Confocal fluorescence images and (b) Statistical analysis of bare GUVs containing SRB in the external solution under different illumination conditions, including λ = 450 nm (14.4 kJ/m²) and λ = 365 nm (5.6 kJ/m² and 32.4 kJ/m²). Data are presented as mean ± s.d. (n =101 for all cases). No vesicle rupture or detectable SRB leakage was observed, confirming that membrane integrity was unaffected by illumination.

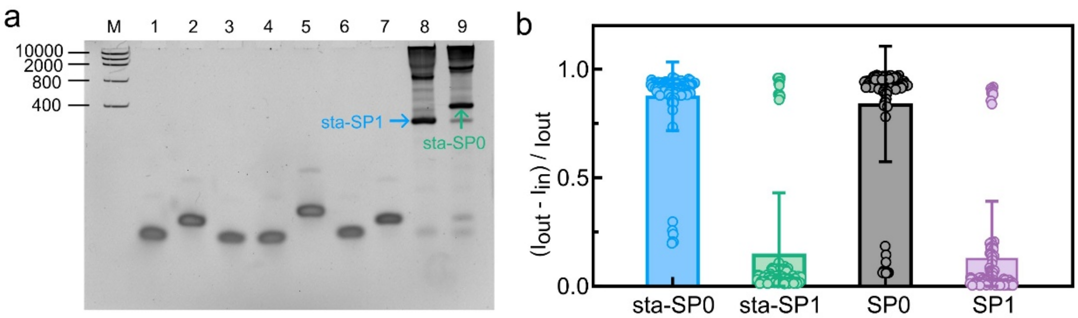

Figure S3. (**a**) 10% Native-PAGE gel confirming the formation of statically-open small nanopores (sta-SP1) and statically-closed nanopores (sta-SP0) that lack azobenzene modification. M: high-range DNA marker, 1: strand S1, 2: strand S2, 3: strand S3, 4: strands S4, 5: strand S5, 6: strand S6, 7: strand lid, 8: sta-SP1, formed by strands S1+S2+S3+S4+S5+S6, 9: sta-SP0, formed by strands S1+S2+S3+S4+S5+S6+lid. (**b**) Influx ratios for GUVs with sta-SP0, sta-SP1, SP0 and SP1. Data represent mean ± s.d. from three independent experiments. n for all four cases is 110.

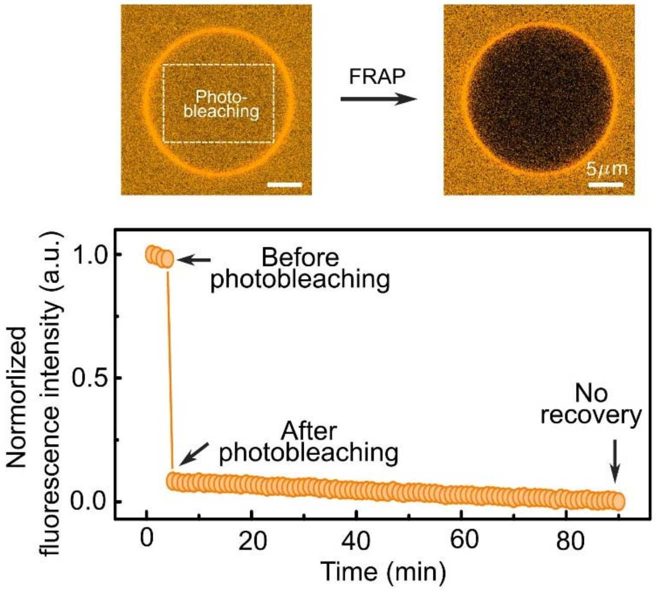

Figure S4. **Closure of SPs upon illumination of visible light, as verified by the FRAP experiment.** Photobleaching of SRB within SP0-bound GUVs after visible light exposure reveals no fluorescence signal recovery.

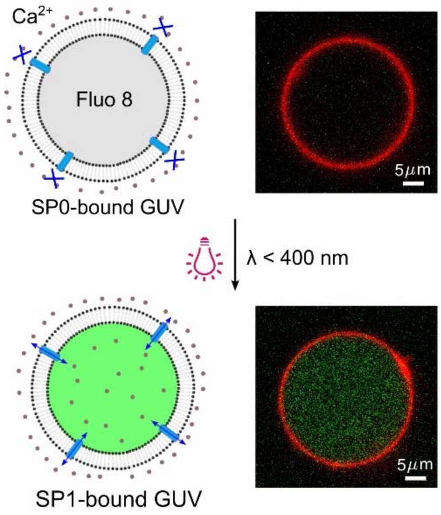

Figure S5. The light-triggered opening of SPs enables Ca^{2+} influx, evidenced by the fluorescence increase of the Ca^{2+}-responsive probe Fluo-8 encapsulated inside the GUV.

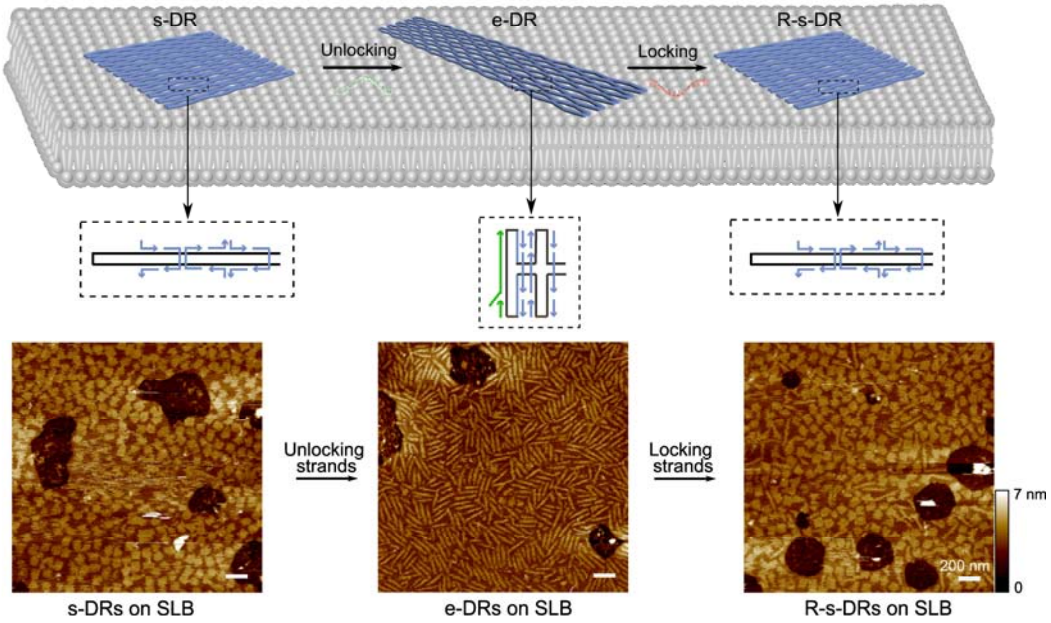

Figure S6. **AFM images of the DRs, transforming among different states on an SLB.** The addition of unlocking strands triggers the reconfiguration from s-DRs to e-DRs, while the addition of locking strands initiates the reconfiguration from e-DRs to R-s-DRs.

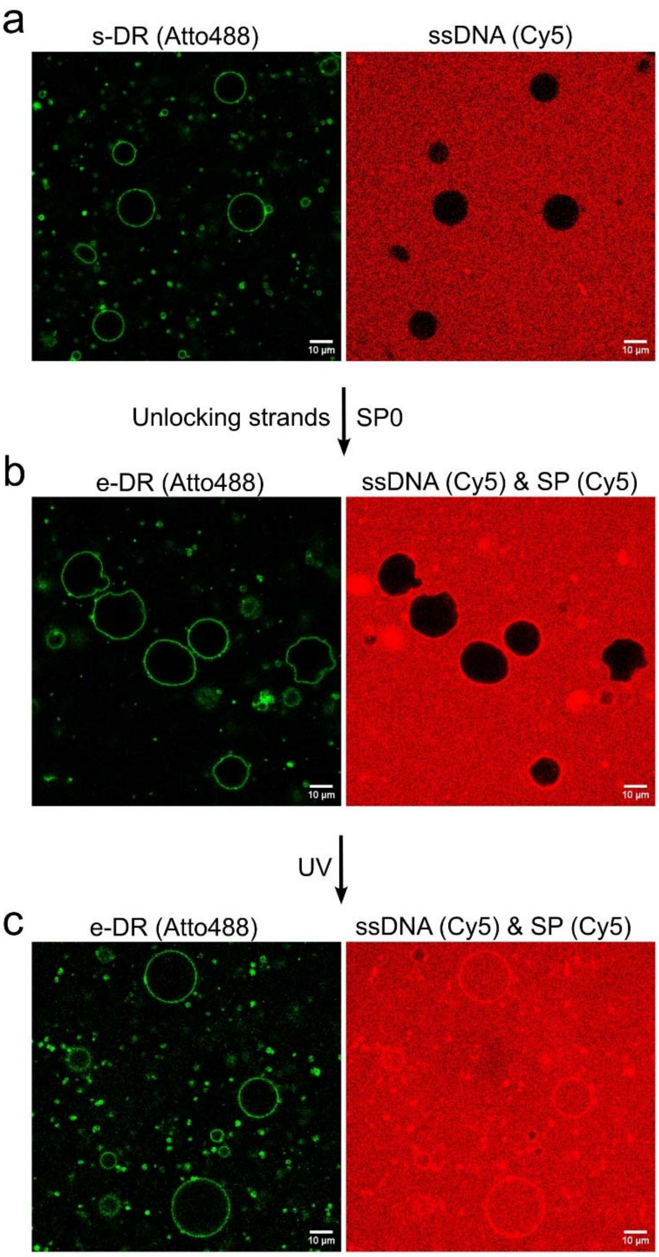

Figure S7. Formation of the LPs, due to the opening of SPs and reversible morphological changes of GUVs. (**a**) No ssDNA transport occurs in s-DR-bound GUVs. (**b**) No ssDNA transport is observed during membrane deformations and the introduction of SP0 to e-DR-bound GUVs. (**c**) Activation of SPs by UV illumination results in GUV shape recovery, formation of LPs, and ssDNA transport into GUVs.

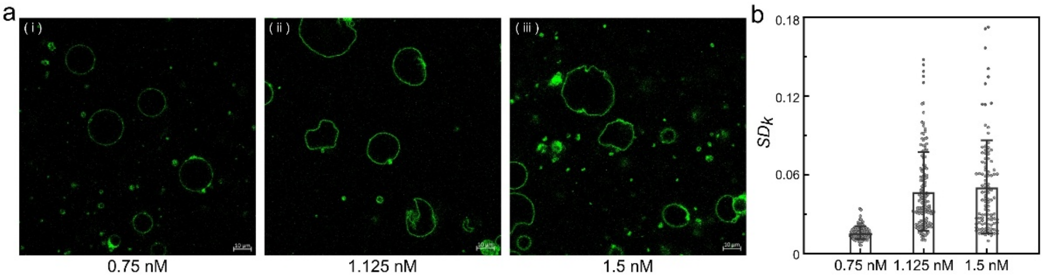

Figure S8. Relationship between DNA raft concentration and membrane deformation under isosmotic conditions. (**a**) Representative confocal images of GUVs incubated with e-DRs at 0.75 nM, 1.125 nM, and 1.5 nM, showing the degree of membrane deformation. (**b**) Corresponding statistical analysis showing the standard deviation of normalized local curvature (SD_k) for each condition. Data represent mean ± s.d. from three independent experiments. n = 107, 152, and 106 for 0.75 nM, 1.125 nM, and 1.25 nM, respectively. At low concentrations (e.g., 0.75 nM), membrane deformation was insufficient, whereas concentrations above the threshold (e.g., 1.125 and 1.5 nM) reliably induced deformation.

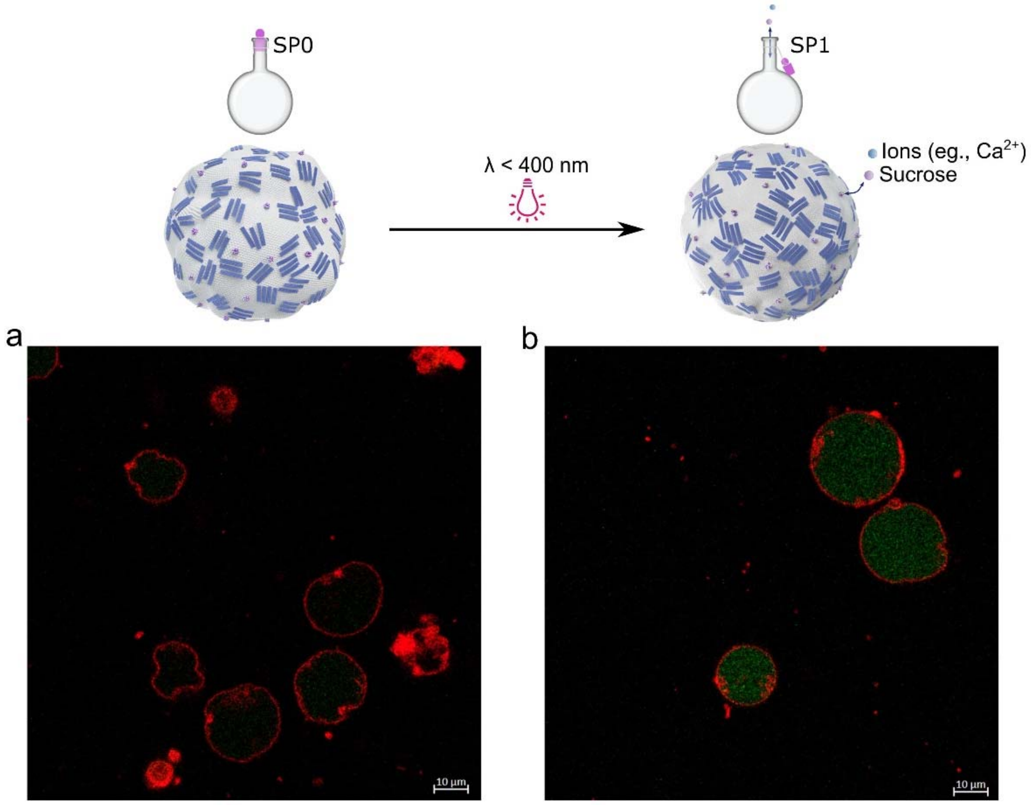

Figure S9. Confocal fluorescence images of GUVs encapsulating Ca^{2+}-responsive fluorophore Fluo-8, showing Ca^{2+} influx through SPs during GUV shape recovery prior to LP formation. (**a**) e-DR-bound GUVs encapsulating Fluo-8 remained non-fluorescent after GUV deformation. As Fluo-8 (~1.1 kDa) is impermeable to SPs (see Fig. S5), fluorescence activation occurs only upon Ca^{2+} influx. (**b**) Upon UV-induced

SP opening, a pronounced fluorescence increase was observed concomitant with GUV shape recovery, indicating Ca^{2+} influx through SPs before LP formation and supporting osmotic equilibration as a prerequisite step. Note that LP formation occurred only after approximately 50 min following this process (see Fig. S10), at which point the influx of larger molecules (e.g., Cy5-ssDNA, ~9 kDa) was detected.

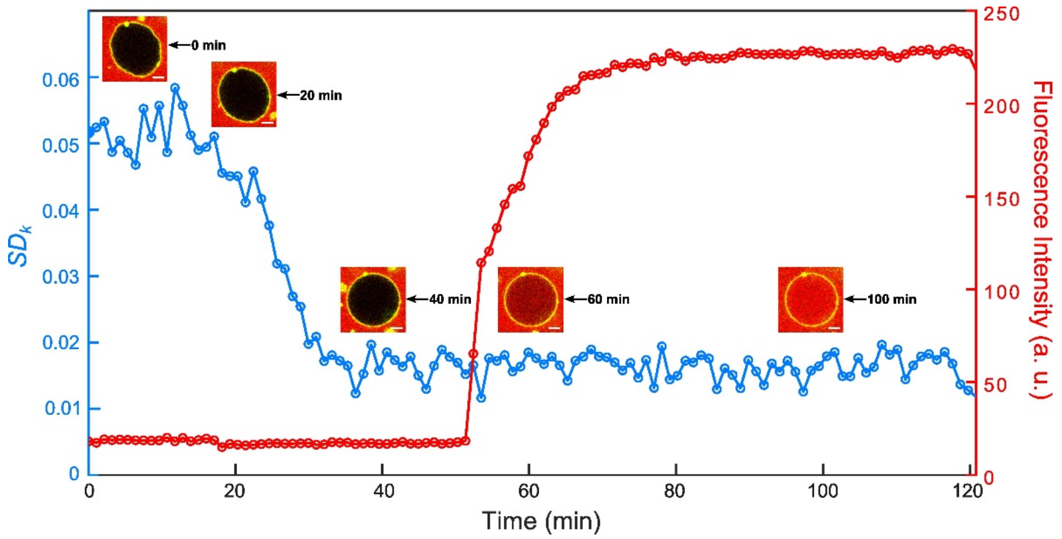

Figure S10. Standard deviation of the normalized local curvature (SD_k) of the GUV trace (blue curve) and fluorescence intensity within the GUV (red curve) recorded over time. Inset: scale bars, 5 μm.

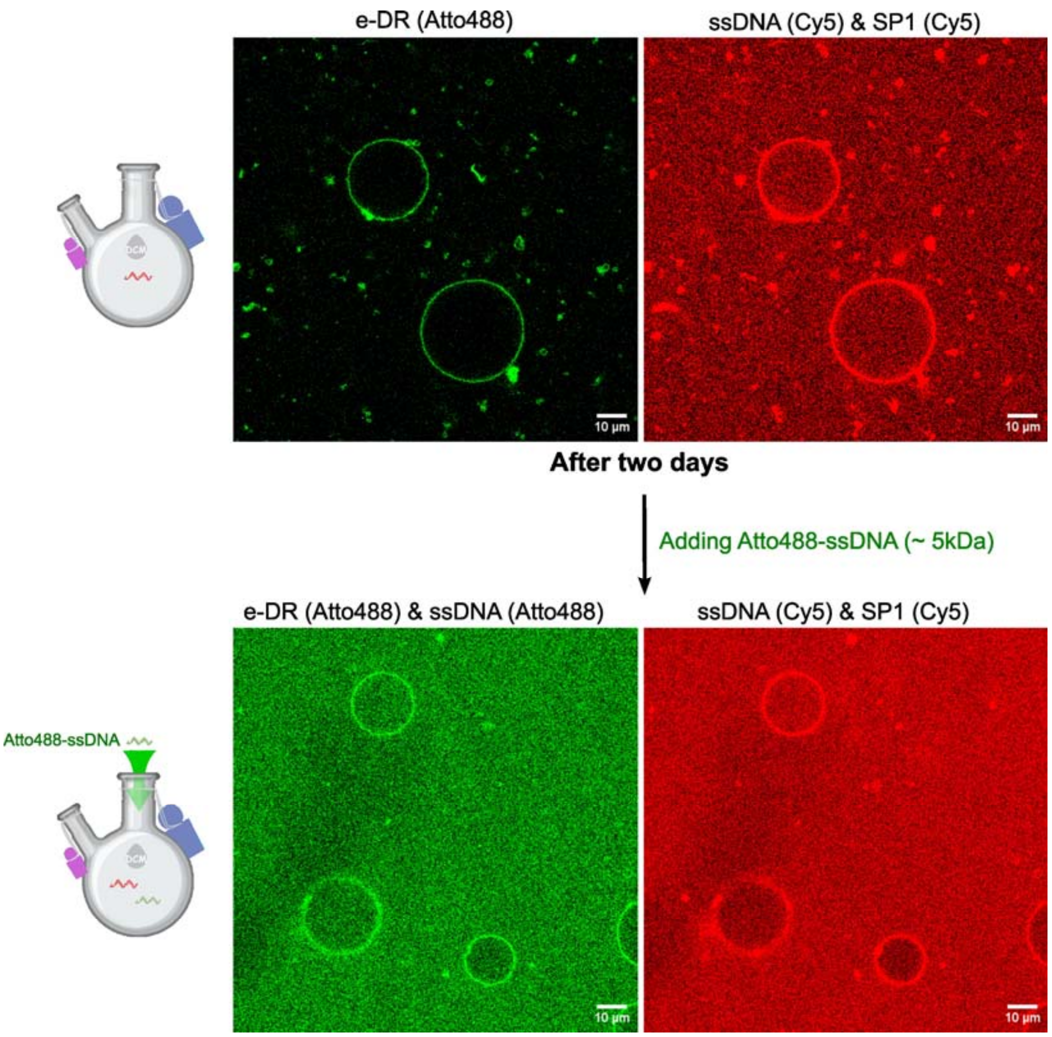

Figure S11. Long-term stability verification of LPs. After two days, Atto488-labeled ssDNA (~5 kDa) can still be transported through LPs into GUVs.

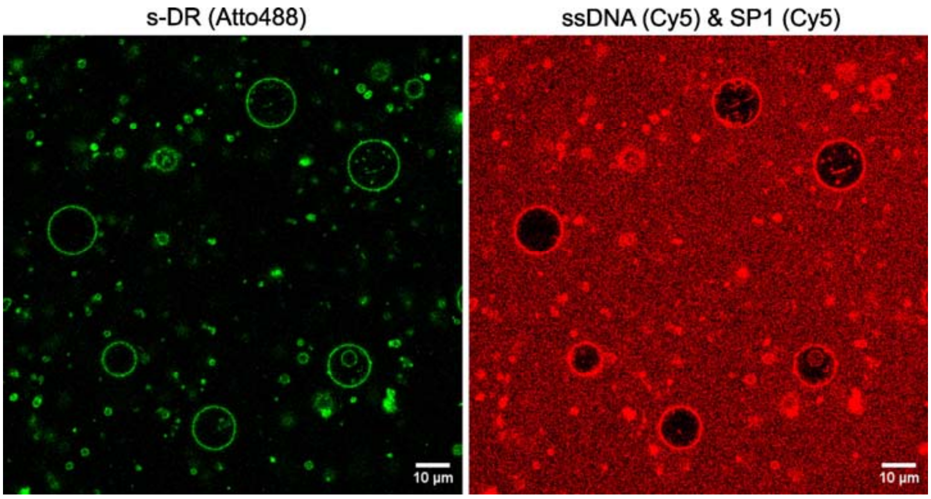

Figure S12. In the presence of SP1, s-DR-bound GUVs show no ssDNA influx.

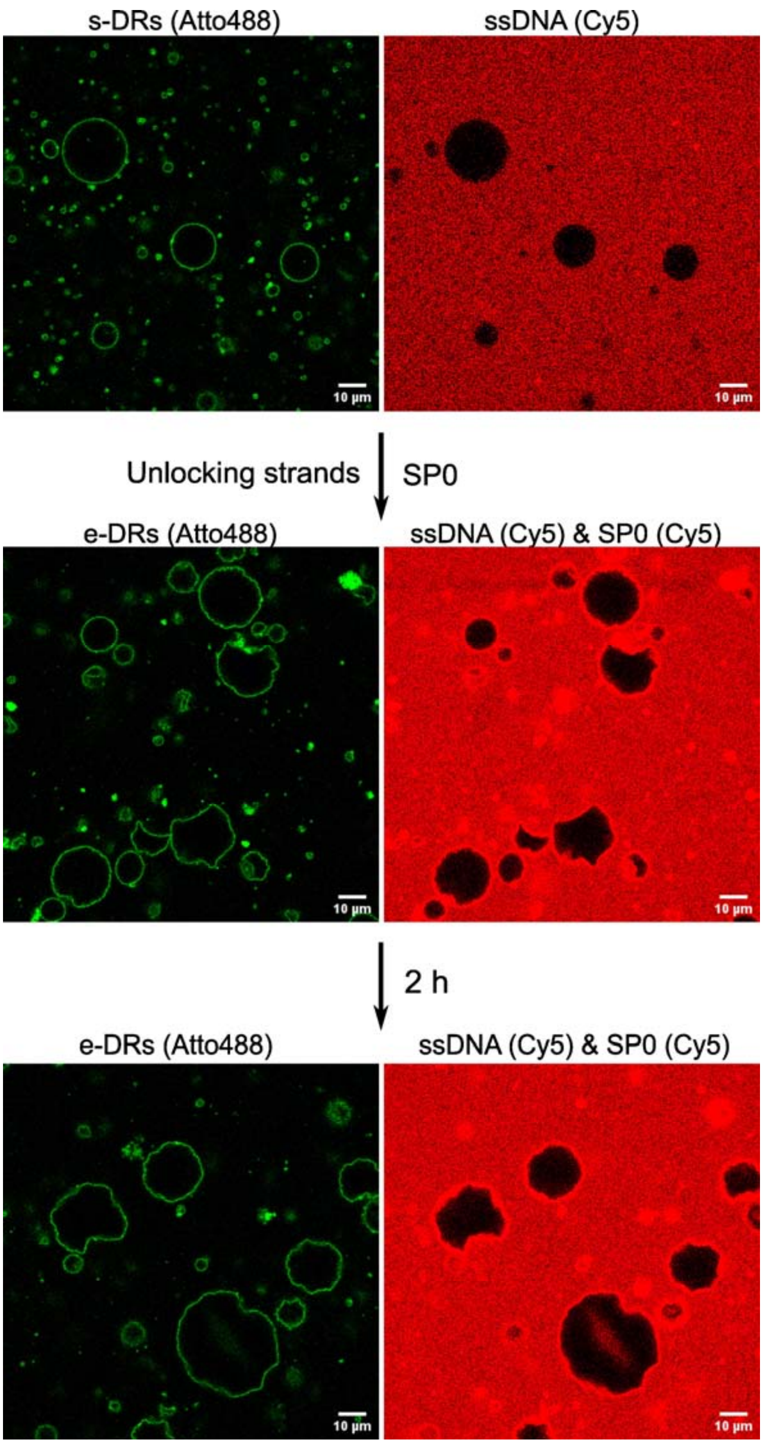

Figure S13. In the presence of SP0, no GUV shape recovery or ssDNA influx are observed, indicating the absence of LPs and no DCM formation.

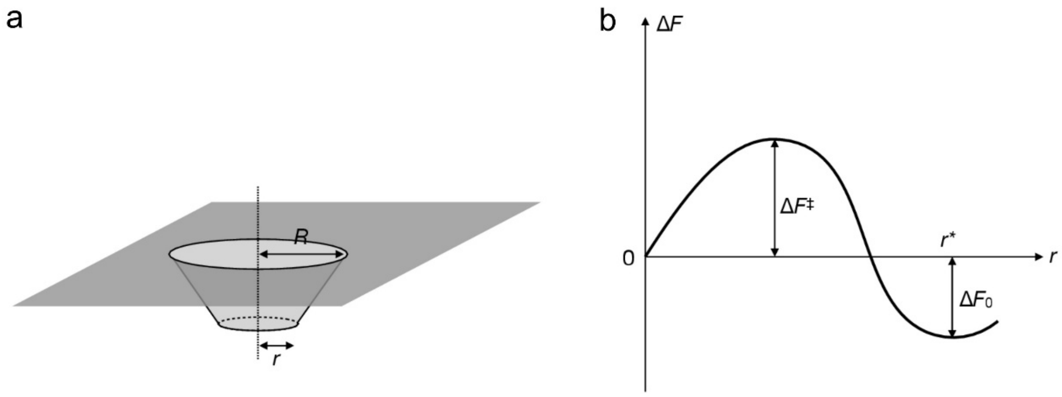

Figure S14. Theoretical framework to qualitatively understand the mechanism of LP formation. (**a**) Sketch of a conical indentation. (**b**) Expected behavior for the free energy difference ΔF due to a pore with radius r. The barrier with height $\Delta F^{\ddagger}$ is due to the competition of gaining area vs. the penalty γ for having to sustain the pore edge. Reducing the local density of DNA rafts releases the pressure, which we expect to yield a minimum corresponding to the stable pore size.

In the experiments, we observe that the lipid membrane ruptures and sustains local openings (LPs) that form during the GUV shape recovery process. These LPs have an average diameter of approx. 15 nm (cf. Fig. 2d and Fig. S17) and appear to be stable for an extended time. Since the lipids are mobile, the DNA rafts cannot sustain in-plane forces as the mechanism underlying LP formation. In Ref. 45, we have shown that the pressure $p(\phi)$ due to the excluded volume of DNA rafts (with packing fraction ϕ) can drive membrane deformation once it exceeds a critical pressure p^*. The reason is that the DNA rafts move in a plane displaced by ℓ (due to the cholesterol anchors) and thus access a slightly larger area, which can be further enlarged through deformation. The pressure can be written as $p(\phi) = p_0 Z(\phi)$ with dimensionless compressibility factor Z (with $Z \to \phi$ in the dilute limit and rising to $Z \simeq 10$ for intermediate packing fractions) and scale factor $p_0 = k_B T/a \sim 10^{-4} k_B T/\text{nm}^2$ (with the area $a = 3800\ \text{nm}^2$ of a single raft). For the critical pressure we have found $p^* \sim \kappa/(\ell R_0) \sim 10^{-3} k_B T/\text{nm}^2 = 10 p_0$ with bending modulus $\kappa = 10 k_B T$ and GUV radius $R_0 = 10\ \mu\text{m}$, which is consistent with the observed onset of deformed GUVs for sufficiently dense DNA rafts.

We now discuss two potential mechanisms for a local membrane perforation along the same lines. First, let us consider a conical indentation with base radius R and pore radius $r = R\sqrt{1 - \cos\alpha}$ assuming that the membrane area is conserved (Fig. S14a). Such an indentation increases the area available to the DNA rafts by $2\pi R \ell \alpha$, where $\frac{\pi}{2} - \alpha$ is the cone's half-angle. Neglecting the cost to bend the membrane implies a free energy change

$$\Delta F = 2\pi r \gamma - p 2\pi R \ell \alpha = 2\pi R[\gamma\sqrt{1 - \cos\alpha} - p\ell\alpha]$$

with γ the edge energy per length. Expanding the cosine for small α, we obtain the critical pressure $p^* \sim \gamma/\ell$ for an indentation to become favorable. Assuming for a

“pure” lipid membrane that the edge is sealed by bending the lipids would imply $\gamma \sim \kappa/d$ with membrane thickness $d \sim 5$ nm leading to a very high critical pressure $p^* \sim 10^4 p_0$. The magnitude is corroborated by simulations that find $\gamma \sim k_B T/\text{nm}$.[1] Although there is evidence that the cholesterol prefers regions with high curvature and thus might reduce γ, we conclude that this effect is not strong enough and that the spontaneous formation of conical pores is suppressed.

Second, opening a pore directly without indentation increases the area available for the DNA rafts by πr^2. Initially, we have $\Delta F \approx \gamma 2\pi r - p(\phi)\pi r^2$ although increasing the area (in particular for several pores) will decrease ϕ and the pressure, and thus the driving force for pore formation. Assuming a constant pressure for a single pore, we estimate the barrier $\Delta F^{\ddagger} \simeq \pi\gamma^2/p \sim (10^4/Z)k_B T$. Under normal circumstances this barrier is prohibitively large.

However, during the recovery of the spherical shape for the GUV, the accessible area again becomes smaller and consequently, the pressure p rises. There is evidence that the DNA rafts are dynamically arrested (vanishing diffusion) so that the local order is locked in (cf. Ref. 45). Moreover, such an arrest is an indication that we approach a jamming transition, for which typically $p \sim (\phi_0 - \phi)^{-\alpha}$ diverges as a power law. We hypothesize that defects further exacerbate the local increase of pressure as they more strongly resist compression, which suggests that in the vicinity of defects the barrier $\Delta F^{\ddagger}$ is lowered even further to the point where pores open spontaneously and are stabilized by the gain $\Delta F_0 < 0$ of free energy (Fig. S14b). From the influx kinetics of proteins, we can estimate the number n of large pores to be on the order of 1-10 per GUV. This is compatible with the four nematic defects that have been reported for jammed hard rods on a sphere.[2] We thus conclude that dynamic arrest in combination with nematic defects lowers the barrier to spontaneous pore formation during the GUV shape recovery. Pores are then stabilized by the entropic gain of increasing the area accessible to the DNA rafts.

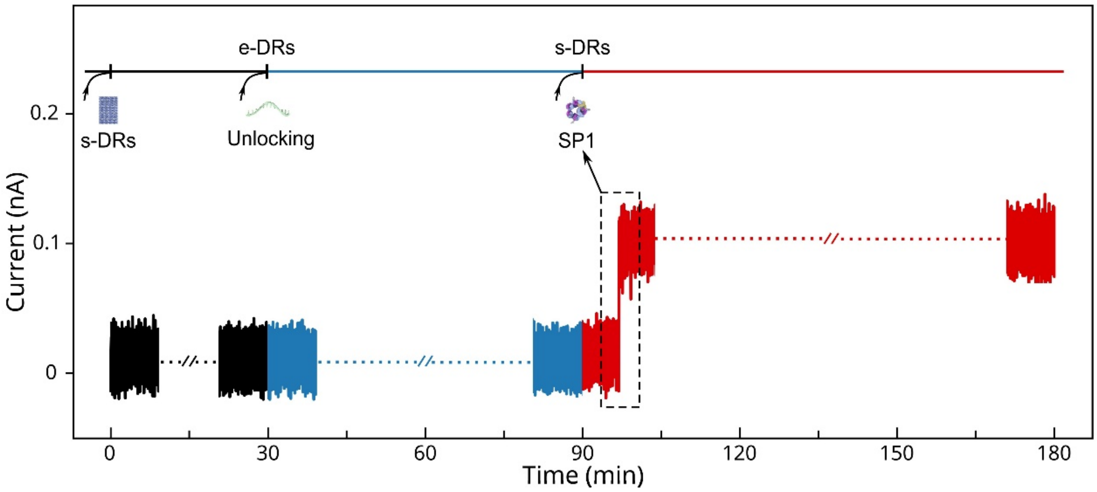

Figure S15. Single-channel recordings of the combined system of DNA rafts and SPs on free-standing planar lipid bilayers. Current traces show sequential steps: s-DR membrane binding (0-30 min, 25 °C) and transformation into e-DRs (30-90 min, 40 °C) upon addition of unlocking strands, recorded under an applied voltage of +50 mV. No channel-forming currents were observed during these steps. Addition of SP1 at 90 min produced an immediate current response, confirming successful membrane insertion. The current remained stable for up to 1.5 hours (90-180 min, 25 °C) without fluctuations, indicating the absence of LP formation. Measurements were performed on 4-cavity microelectrode array (MECA) chips (Ionera, Germany) using an Orbit Mini platform (Nanion Technologies, Germany). Neither the initial binding of s-DRs to the planar lipid bilayer nor their transition to e-DRs generated channel-forming currents. In contrast, the addition of SPs immediately produced stable current signals, confirming their successful membrane insertion. These currents remained unchanged for over 1.5 h, indicating that LP formation does not occur in the planar lipid bilayer. This result highlights the critical role of membrane dynamics in LP formation, which relies on the coupling between DNA raft reshaping and GUV membrane deformation, with SP1-assisted GUV shape recovery being essential for initiating LP formation and the associated molecular transport.

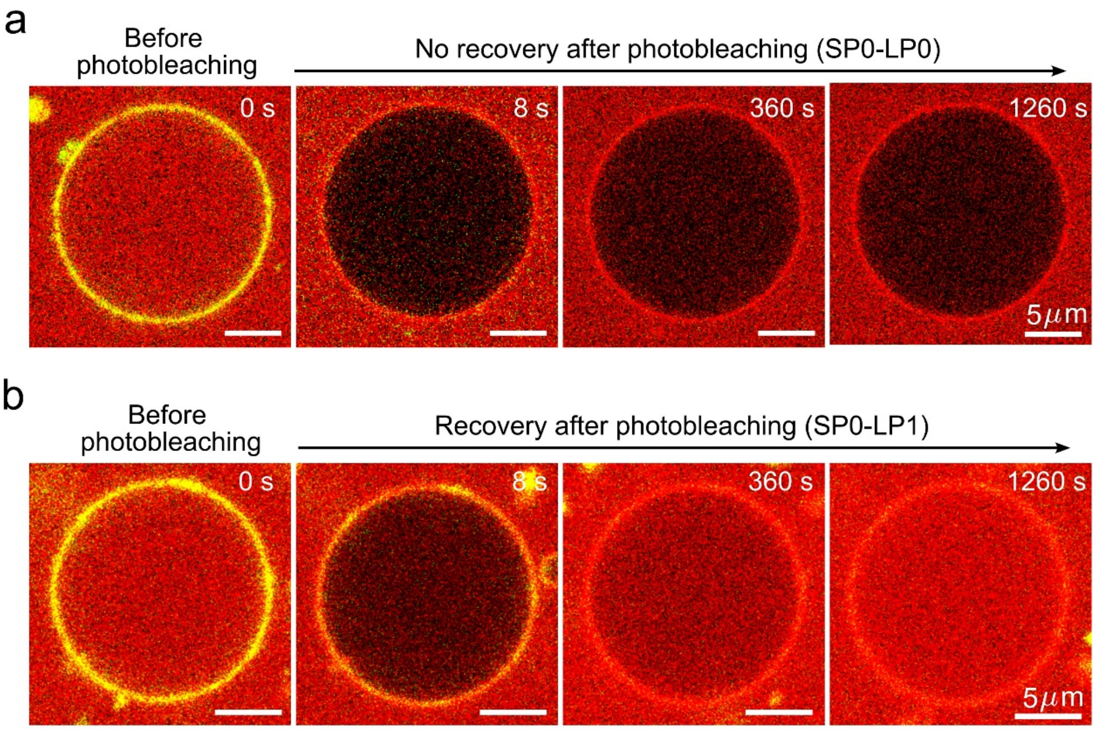

Figure S16. Verification of LP sealing using FRAP experiments. (**a**) Closure of LPs by adding locking strands. Photobleaching of ssDNA-Cy5 within the DCM (SP0-LP0) using FRAP results in no fluorescence signal recovery. (**b**) In the absence of locking strands, photobleaching of ssDNA-Cy5 within the DCM (SP0-LP1) using FRAP shows a rapid fluorescence signal recovery.

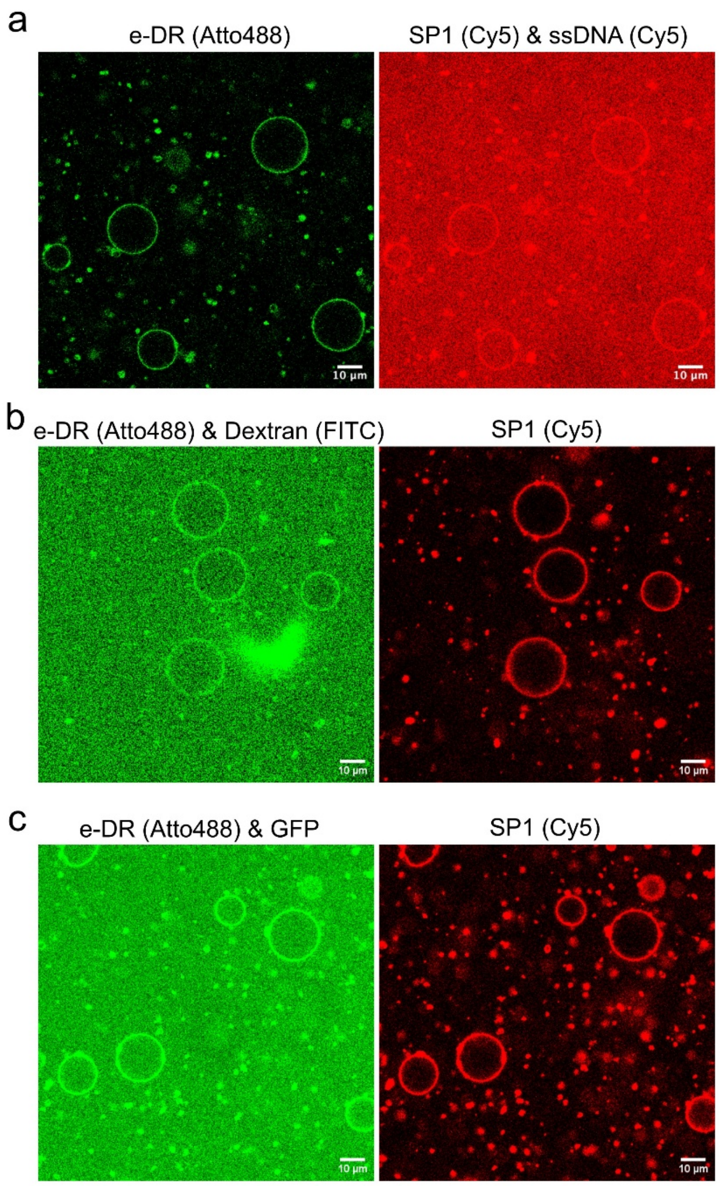

Figure S17. **Confocal images of DCMs (SP0-LP1) in different influx experiments.** (**a**) Cy5-labeled ssDNA (9 kDa), (**b**) FITC-labeled dextran (20 kDa), and (**c**) GFP (27 kDa).

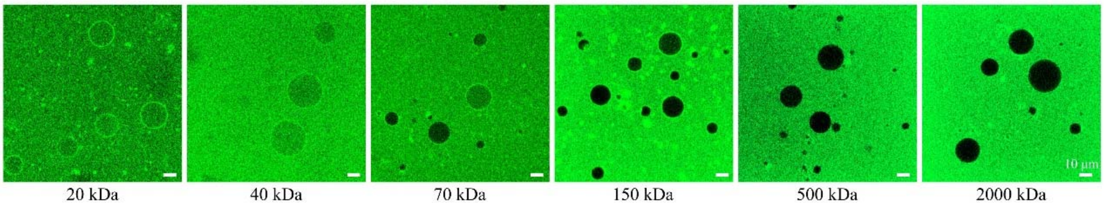

Figure S18. Representative confocal fluorescence images showing the influx of Dextran-FITC of different molecular weights. Scale bars, 10 μm.

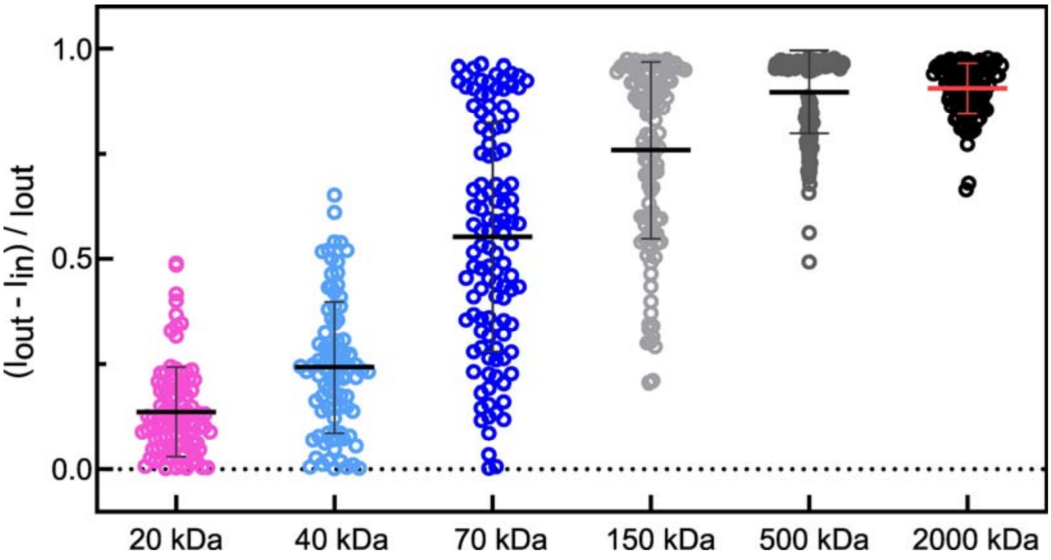

Figure S19. Statistics of the normalized fluorescence intensity difference for dextran of different molecular weights. Data represent mean ± s.d. from three independent experiments: 20 kDa dextran, n = 112; 40 kDa dextran, n = 114; 70 kDa dextran, n = 114; 150 kDa dextran, n = 117; 500 kDa dextran, n = 120; 2000 kDa dextran, n = 114.

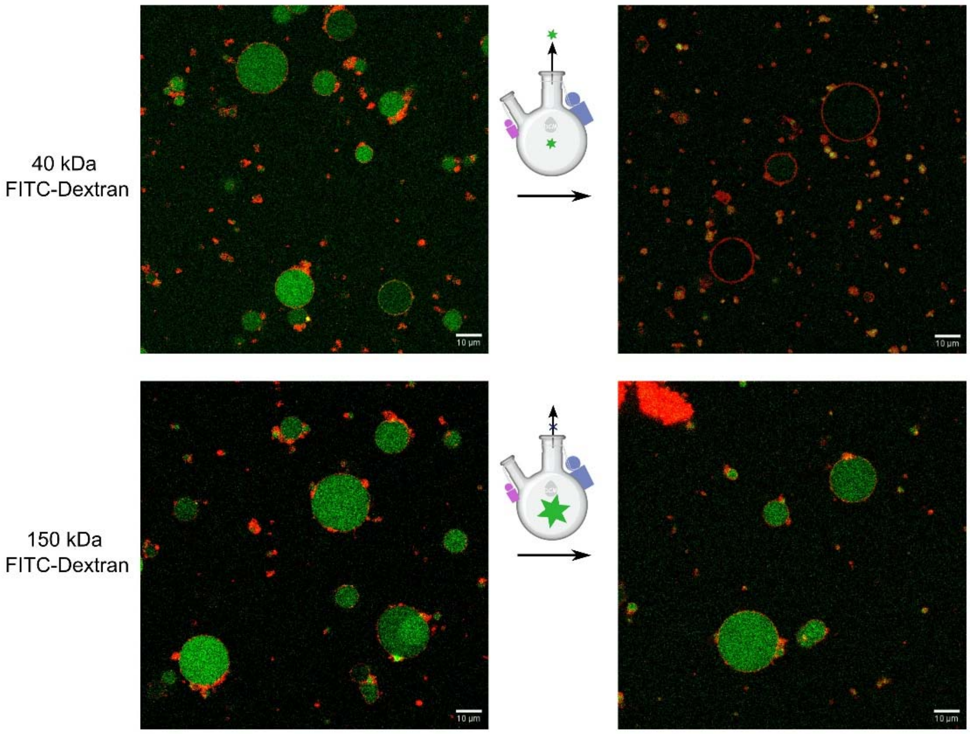

Figure S20. Size-selective molecular efflux of DCMs. DCMs were prepared with GUVs pre-encapsulating 40 kDa and 150 kDa FITC-dextran. Confocal fluorescence images show that at the SP1-LP1 state, 40 kDa FITC-dextran completely diffused out of the DCMs, whereas 150 kDa dextran remained confined inside, demonstrating size-dependent efflux behavior.

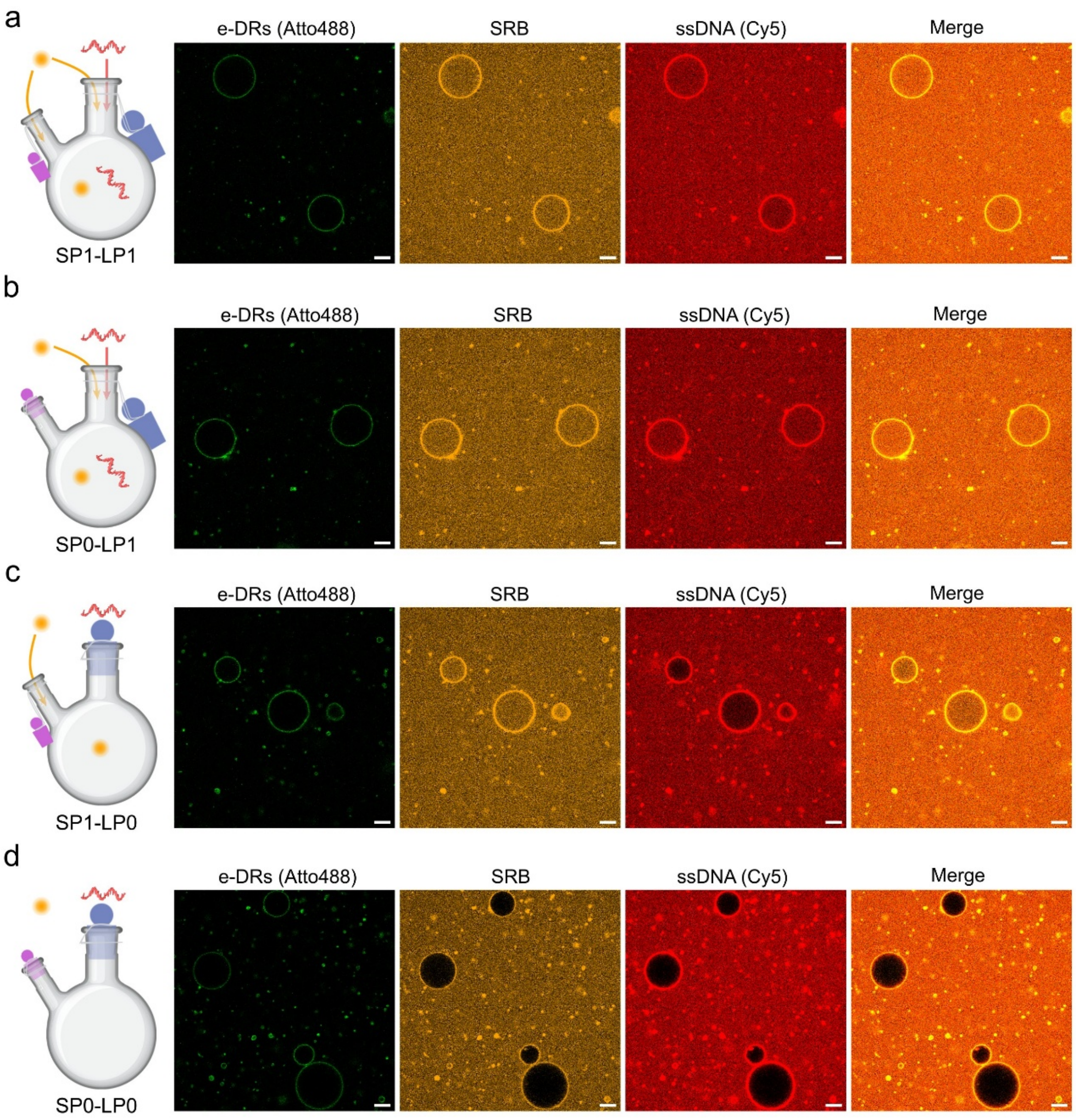

Figure S21. **Confocal images showing the transport of SRB and Cy5-ssDNA into DCMs at different states.** (**a**) SP1-LP1, (**b**) SP0-LP1, (**c**) SP1-LP0, and (**d**) SP0-LP0. Scale bars, 10 μm.

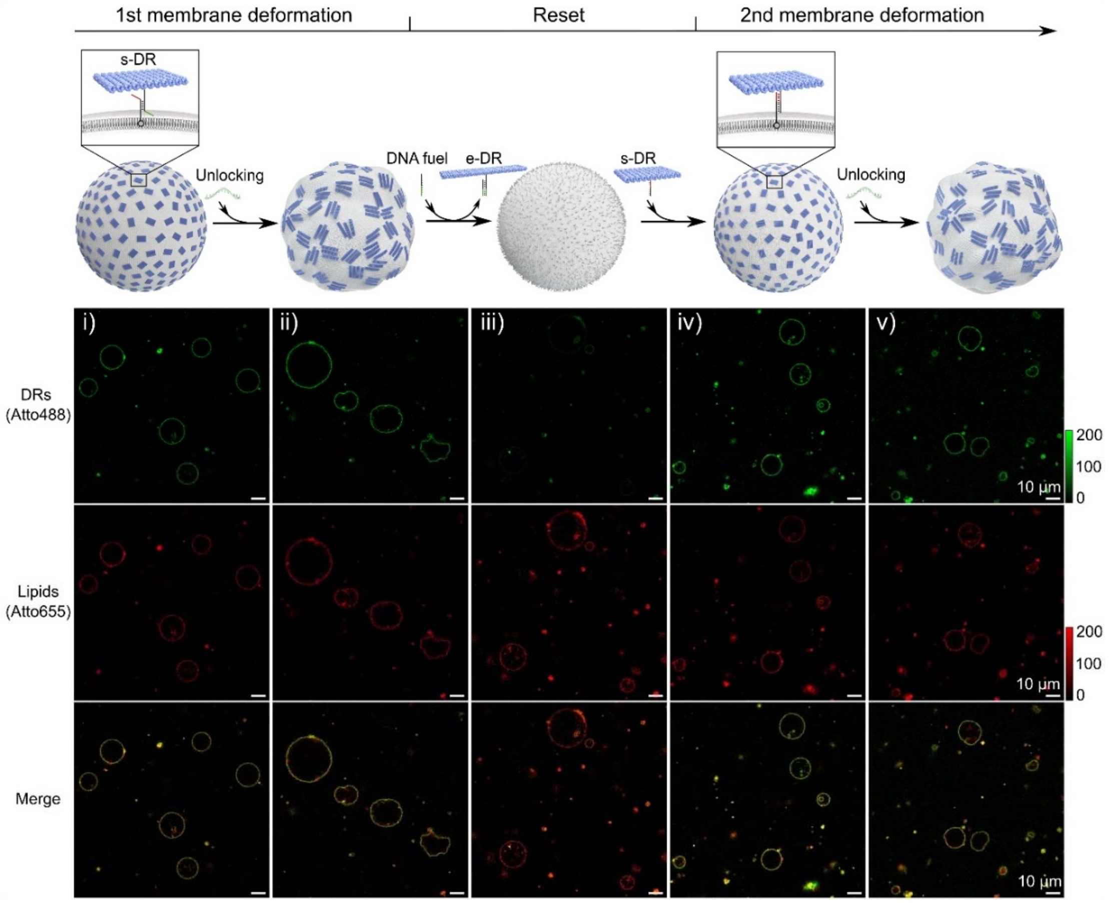

Figure S22. Schematics and confocal fluorescence images illustrating the detachable raft strategy and system reset. (**i**) s-DRs with toehold-modified cholesterol linkers were bound to GUV membranes. (**ii**) Addition of unlocking strands at 40 °C for 2 h induced GUV deformation. (**iii**) Addition of DNA fuel strands triggered detachment of e-DRs from the membrane via toehold-mediated strand displacement at 37 °C for 2 h, confirmed by a marked fluorescence decrease in the DR channel (Atto488), while cholesterol linkers remained on the GUVs. (**iv**) Resupply of s-DRs carrying linkers complementary to the cholesterol anchors restored membrane fluorescence, confirming successful membrane rebinding. (**v**) Subsequent addition of unlocking strands induced GUV deformation, demonstrating the feasibility of the detachable raft strategy and system reset.

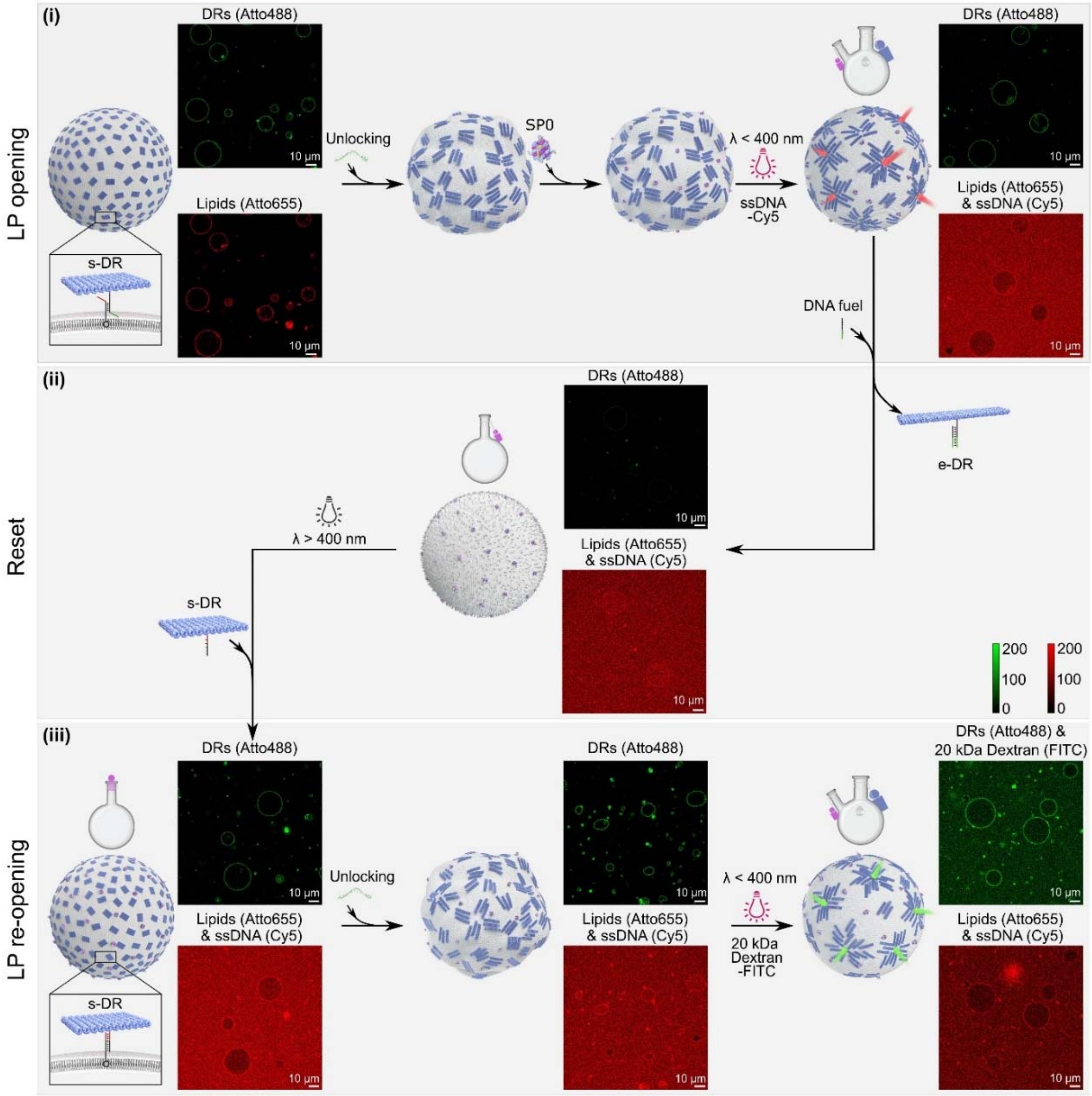

Figure S23. Schematics and confocal fluorescence images illustrating LP opening, system reset, and LP re-opening. (**i**) s-DRs with toehold-modified cholesterol linkers were bound to GUV membranes. Addition of unlocking strands triggered the transformation of s-DRs to e-DRs, inducing GUV deformation. Subsequent SP opening by UV light and GUV shape recovery led to LP formation, confirmed by the influx of Cy5-labeled ssDNA (~9 kDa, red channel). (**ii**) e-DRs were detached from the membrane by adding DNA fuel strands via toehold-mediated strand displacement. SPs were then closed by visible light, resealing the GUV and resetting the system. Freshly supplied s-DRs were attached to the membrane. Repeating the steps in (**i**) led to LP re-opening, confirmed by the influx of FITC-labeled Dextran (20 kDa, green channel).

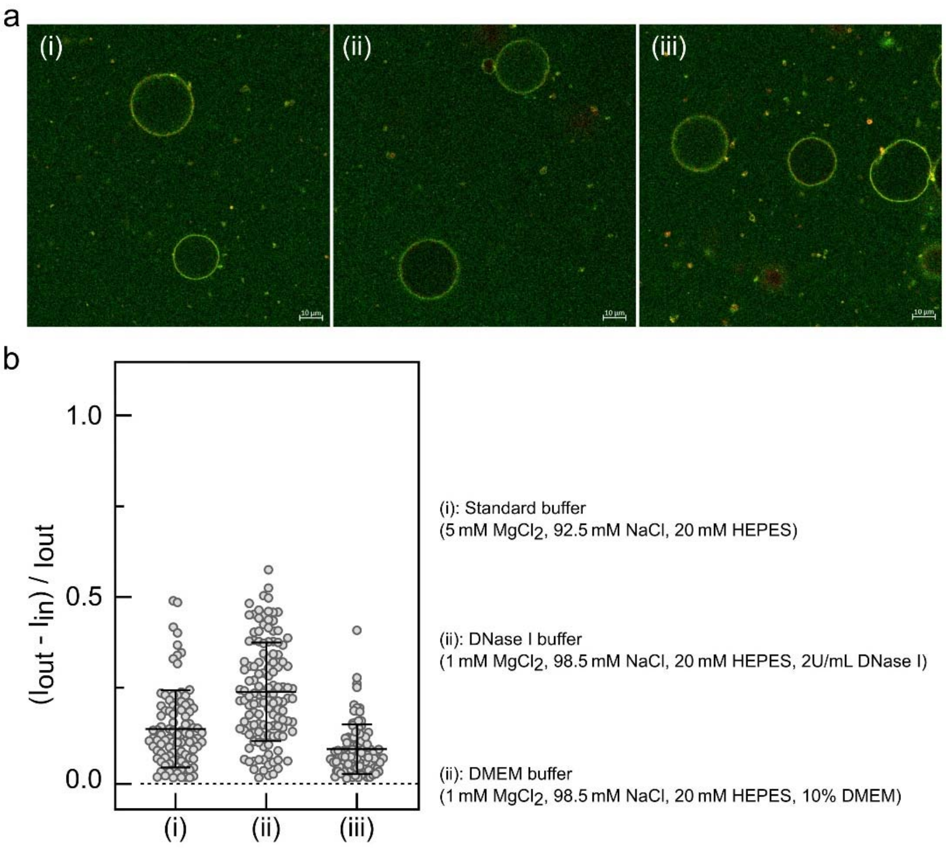

Figure S24. DCM stability and functionality under physiologically relevant conditions. (a) Representative confocal fluorescence images of DCMs showing the influx of 20 kDa FITC-dextran, used as a functional indicator of pore activity. (b) Quantitative analysis of dextran influx efficiency in (i) standard buffer (6 mM Mg^{2+}), (ii) DNase I-containing buffer (2 U/mL, 1 mM Mg^{2+}), and (iii) 10% DMEM shows no significant differences, confirming the structural integrity and functional robustness of DCMs under physiologically relevant conditions. The entire stability test lasted approximately 8 h, comprising DNA raft attachment onto GUV membranes (4 h at room temperature), addition of unlocking strands to induce the transition from s-DRs to e-DRs and subsequent GUV deformation (2 h at 40 °C), UV illumination to open small pores (SP1) and restore GUV morphology, followed by large pore (LP1) formation and associated dextran influx (2 h at room temperature). Data represent mean ± s.d. from three independent experiments. n = 112, 129, and 101 for buffers (i-iii), respectively.

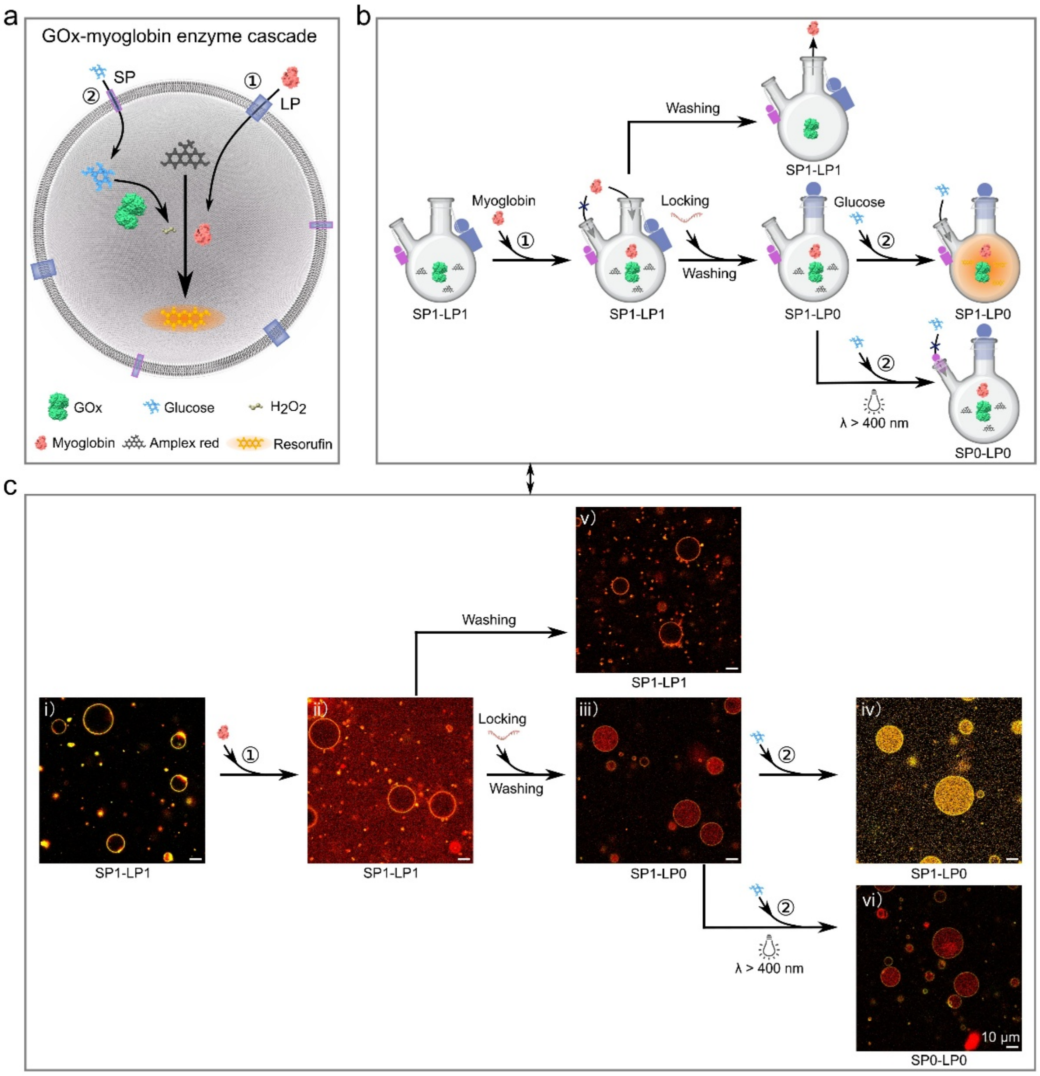

Figure S25. Spatiotemporally controlled GOx-myoglobin enzyme cascade within the DCM. (**a**) Illustration of the GOx-myoglobin enzyme cascade regulated by stepwise transport of reactants. GOx (~160 kDa) converts glucose and oxygen into H_2O_2, which myoglobin (~17 kDa) subsequently uses to oxidize Amplex Red into fluorescent resorufin inside the GUV. (**b**) Schematic and (**c**) confocal fluorescence images of the spatiotemporally controlled cascade process. DCMs pre-encapsulating GOx at the SP1-LP1 state were first prepared, allowing Amplex Red to diffuse in (i). Cy5-labeled myoglobin was then transported into the DCM through LP1, confirmed by Cy5 fluorescence (ii). LPs were subsequently closed to prevent myoglobin leakage after removal of exterior residual myoglobin (iii). Glucose, transported through the remaining open SPs, initiated the cascade, producing resorufin (iv). If LPs were not closed, leakage of myoglobin reduced the internal Cy5 signal (v). Switching the DCM to the SP0-LP0 state blocked glucose entry, resulting in no fluorescence (vi). DRs (Atto488, green), myoglobin and SPs (Cy5, red), and resorufin (orange).

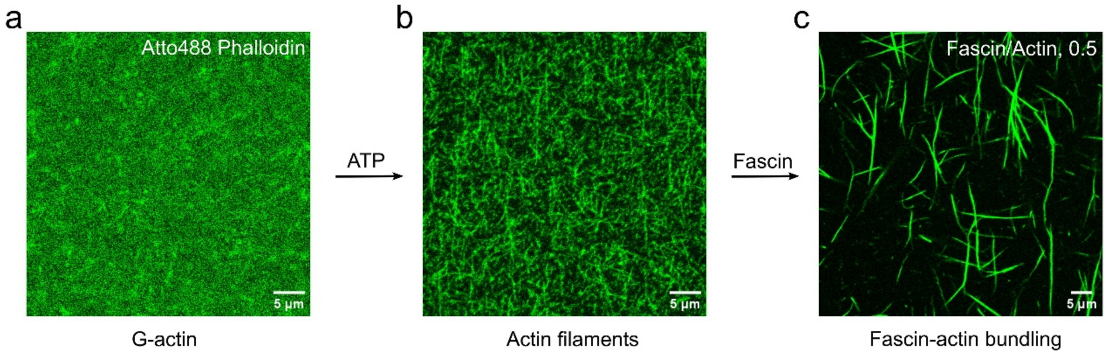

Figure S26. **Actin polymerization and bundling in bulk solutions.** (**a**) Confocal image of 1 μM G-actin in a buffer, containing 5 mM Tris-HCl, 0.2 mM ATP, 1 mM DTT, 100 mM KCl, and 2 mM $MgCl_2$, at pH 8.0. (**b**) Actin polymerization following the addition of 1 mM ATP. (**c**) Actin bundling induced by the addition of 0.5 μM fascin. The actin filaments and fascin-actin bundles are stained with Atto488 phalloidin.

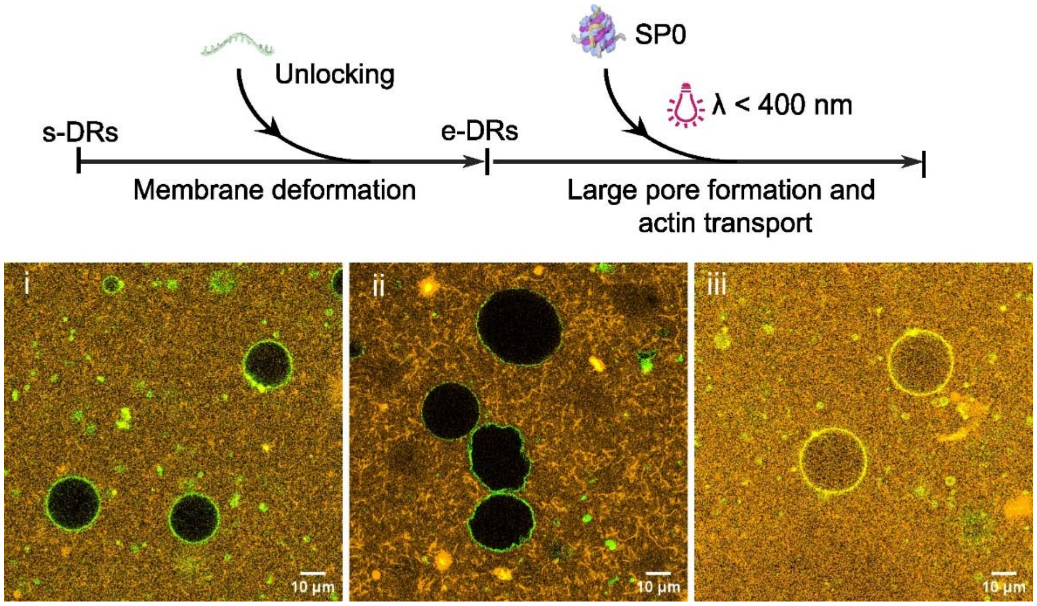

Figure S27. Transport of rhodamine-labeled G-actin (~42 kDa) into DCMs through LPs.

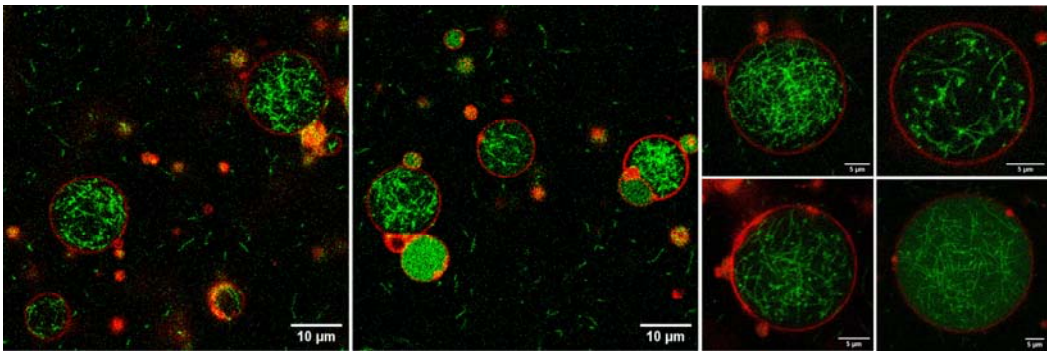

Figure S28. Large field-of-view confocal fluorescence microscopy images of actin polymerization within DCMs after the ATP delivery.

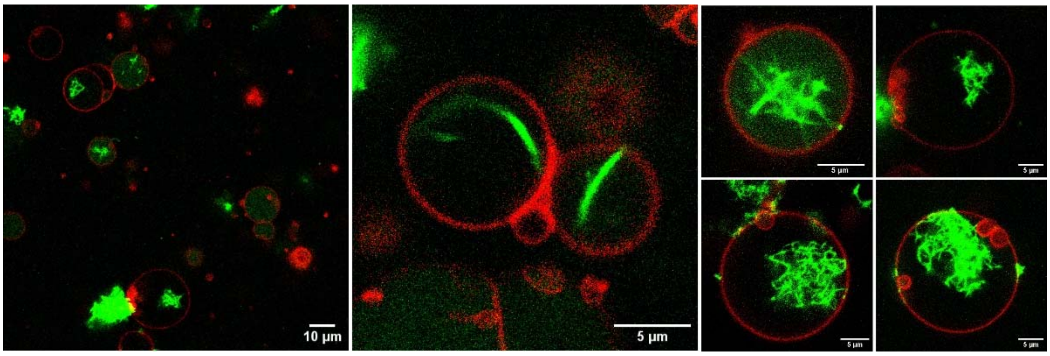

Figure S29. Large field-of-view confocal fluorescence microscopy images of actin filament bundling within DCMs following the transport of fascin.

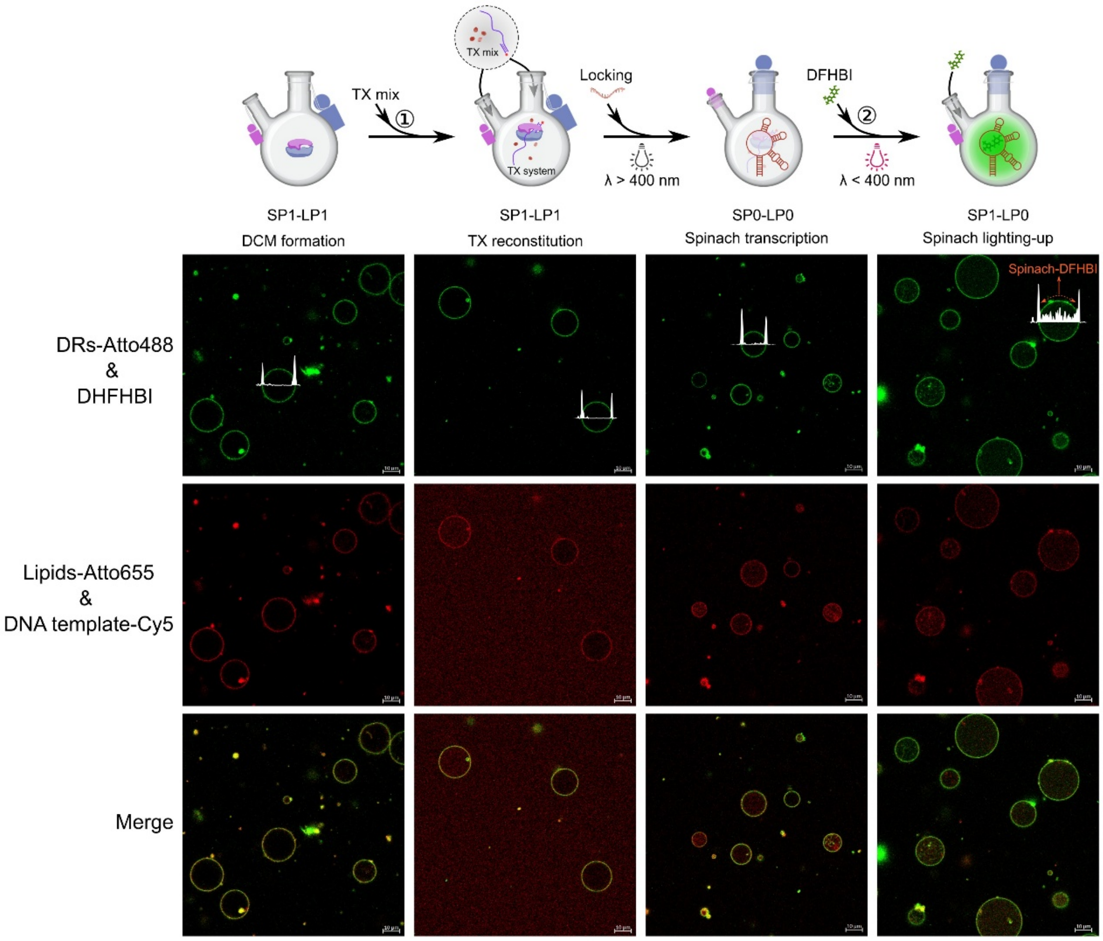

Figure S30. Large field-of-view confocal fluorescence images showing on-demand cell-free RNA transcription and fluorescence visualization within DCMs. Representative green-channel fluorescence profiles (from DRs-Atto488 and DFHBI fluorogen) reveal the sequential transcription of Spinach RNA aptamers and their fluorescence activation within individual DCMs.

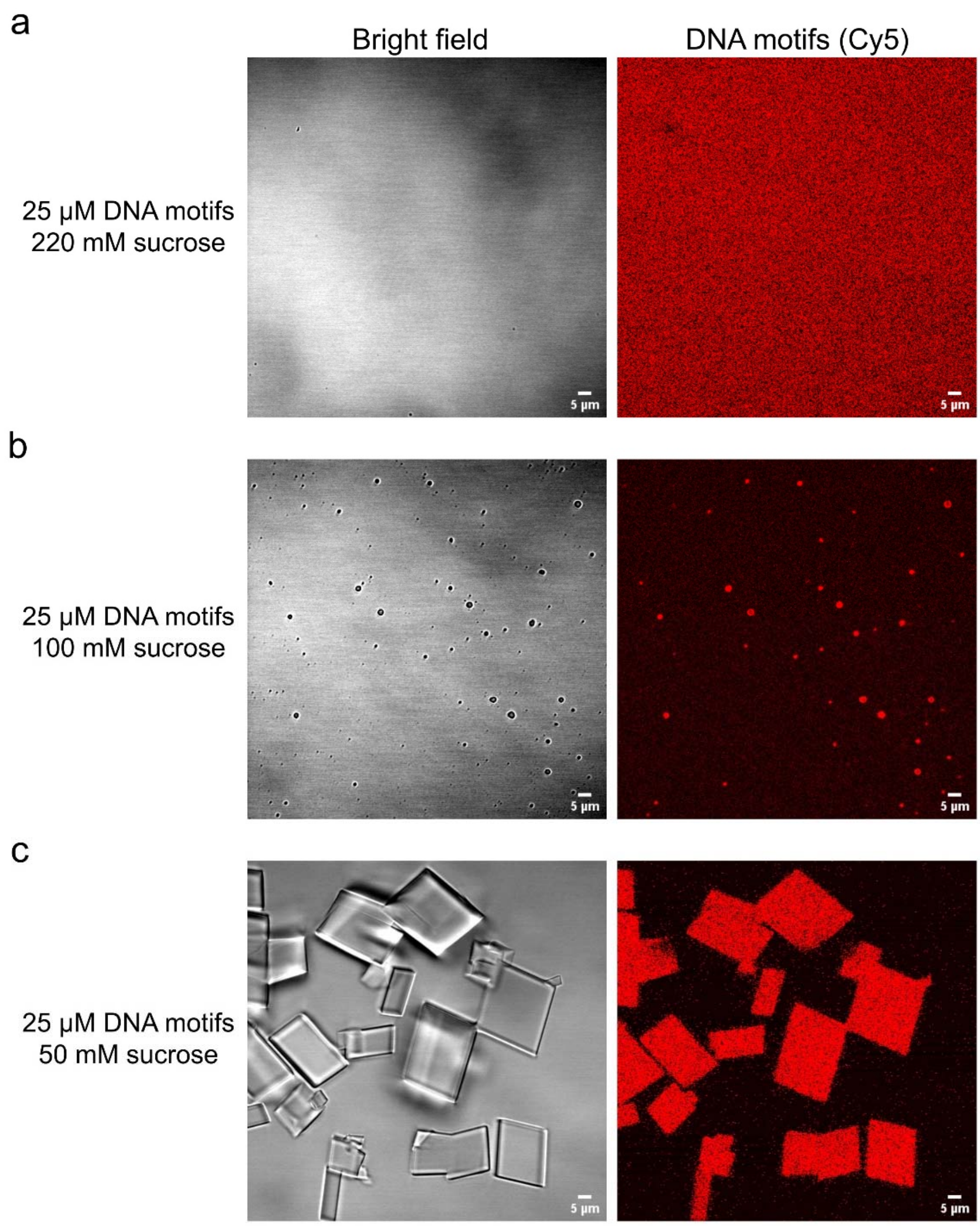

Figure S31. The effect of sucrose concentration on DNA crystallization in 1× TE buffer containing 10 mM Mg^{2+}. The optimal sucrose concentration is below 50 mM.

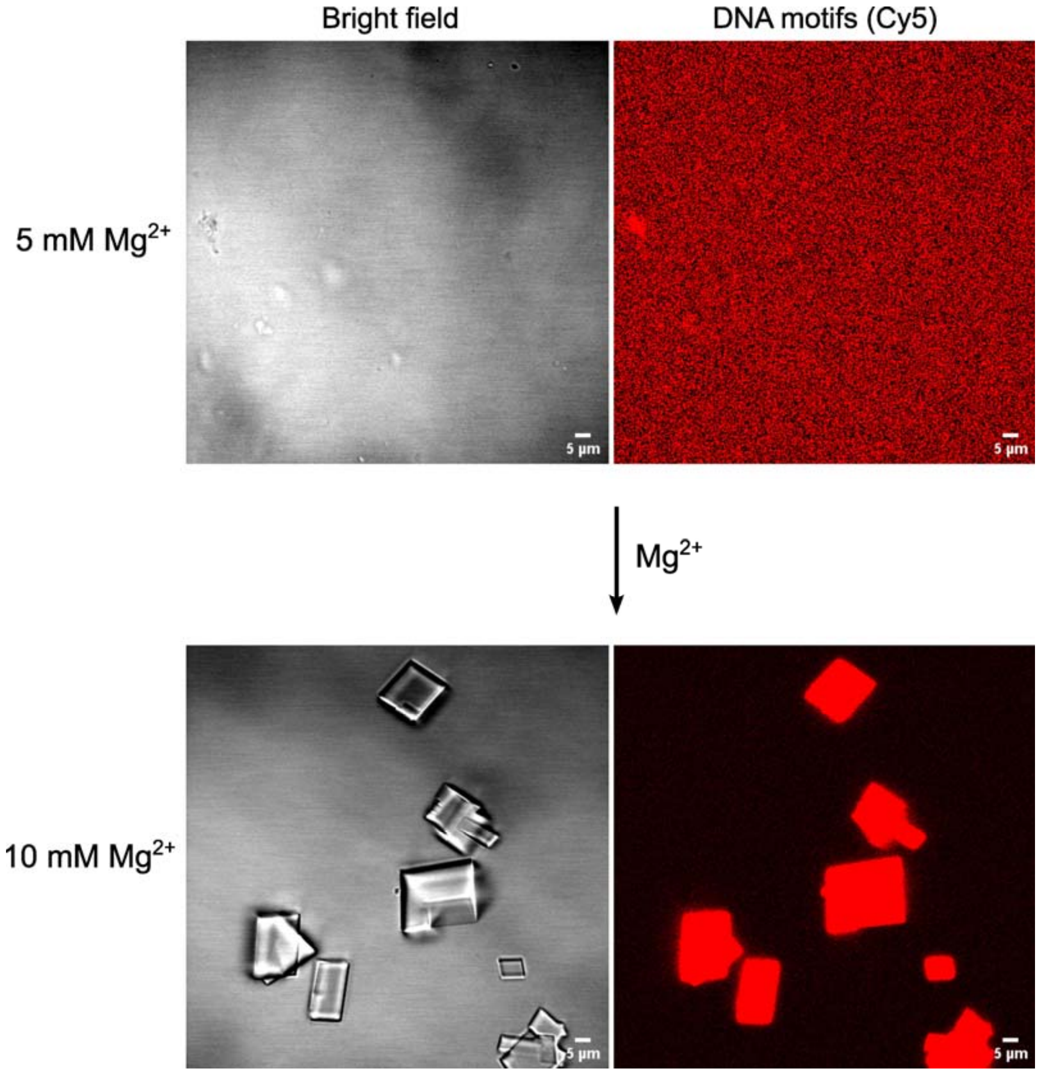

Figure S32. Stepwise control of DNA crystallization by increasing Mg^{2+} concentration from 5 mM to 10 mM.

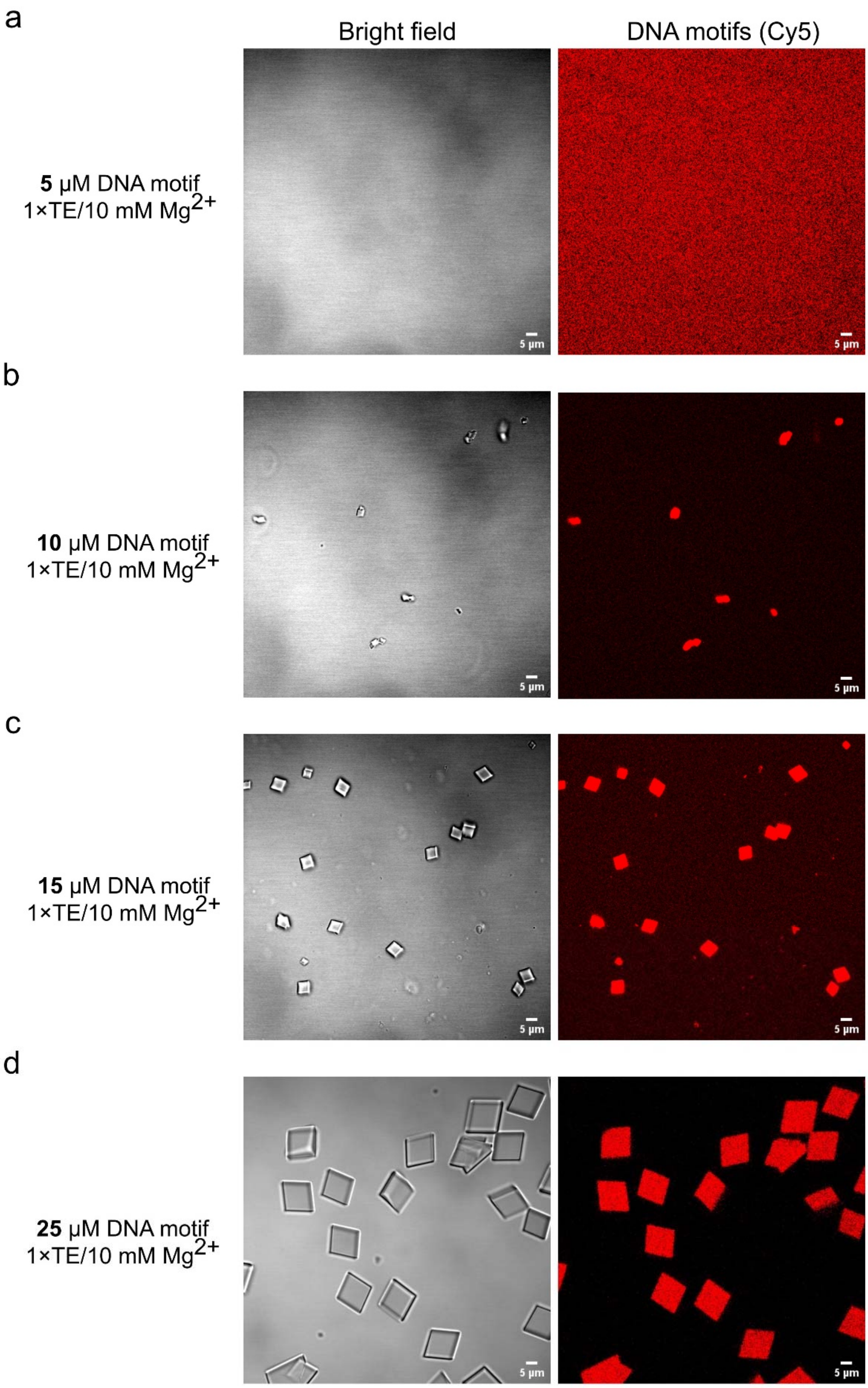

Figure S33. The influence of DNA motif concentration on DNA crystallization in 1× TE buffer containing 10 mM Mg^{2+}. The minimum DNA motif concentration required for crystallization is 10 µM.

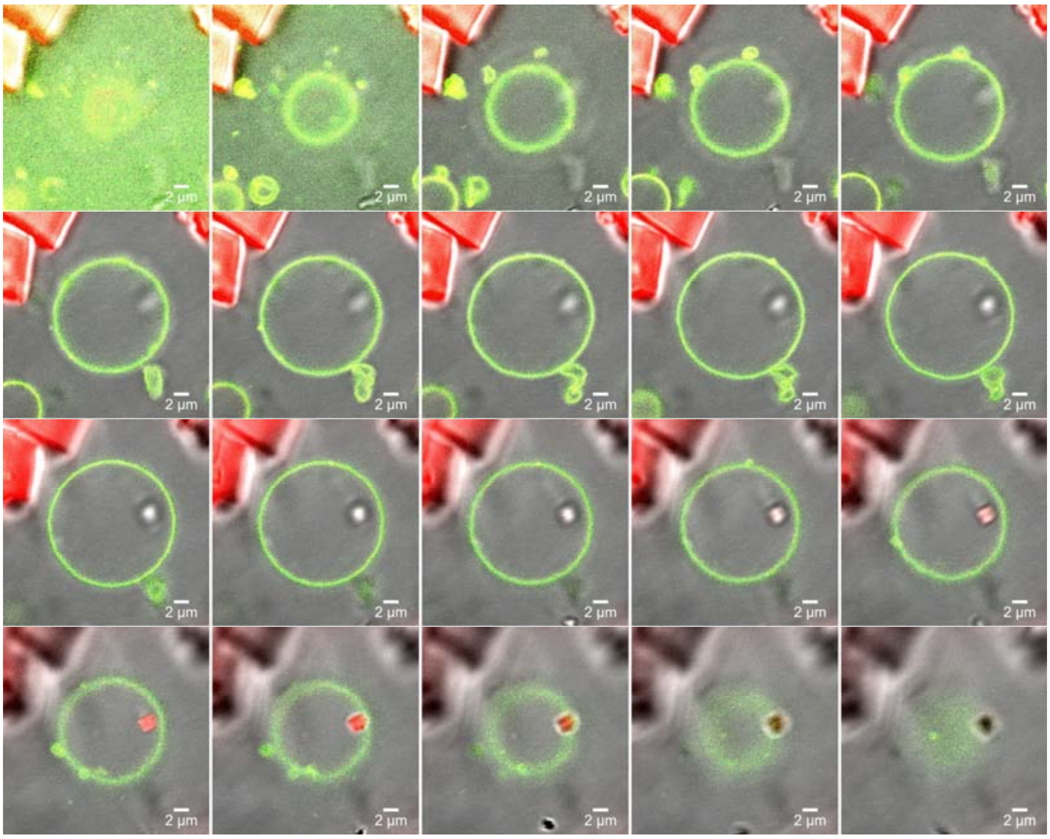

Figure S34. Confocal Z-stack images of a DCM containing a single DNA crystal, presented in 20 μm slices.

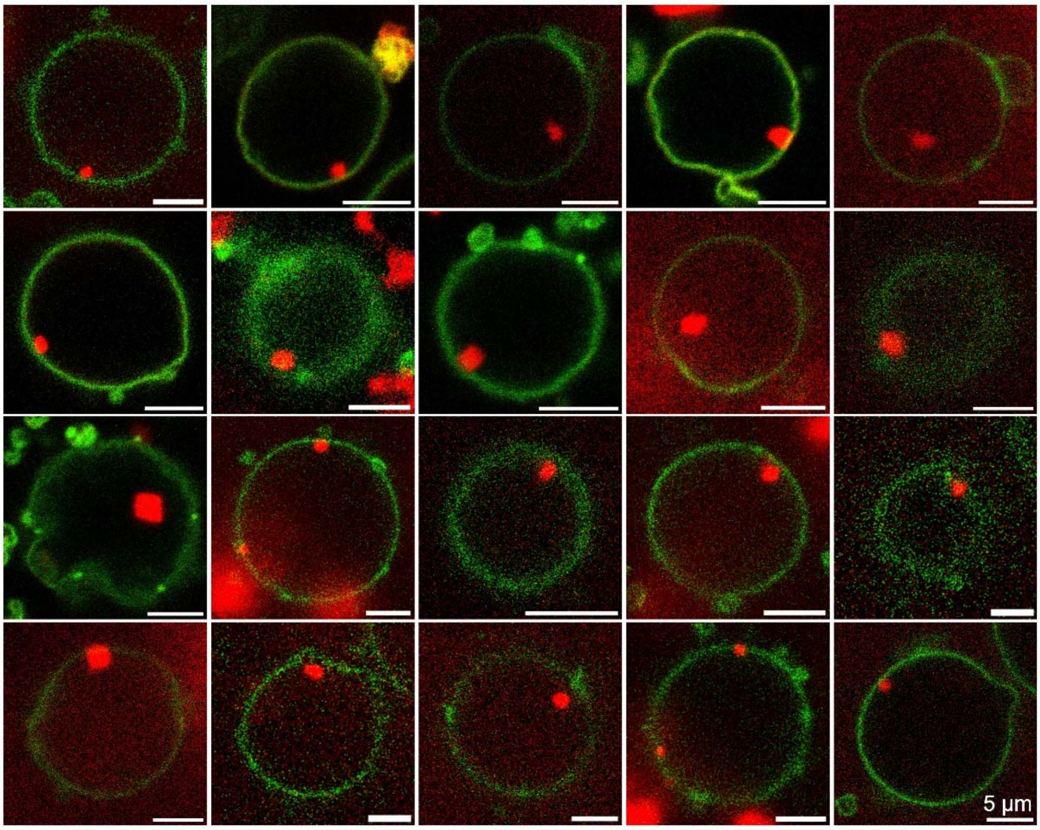

Figure 35. Representative confocal images showing DNA crystals grown within DCMs. DNA crystals of similar size and geometry preferentially localize near the inner surfaces of DCMs.

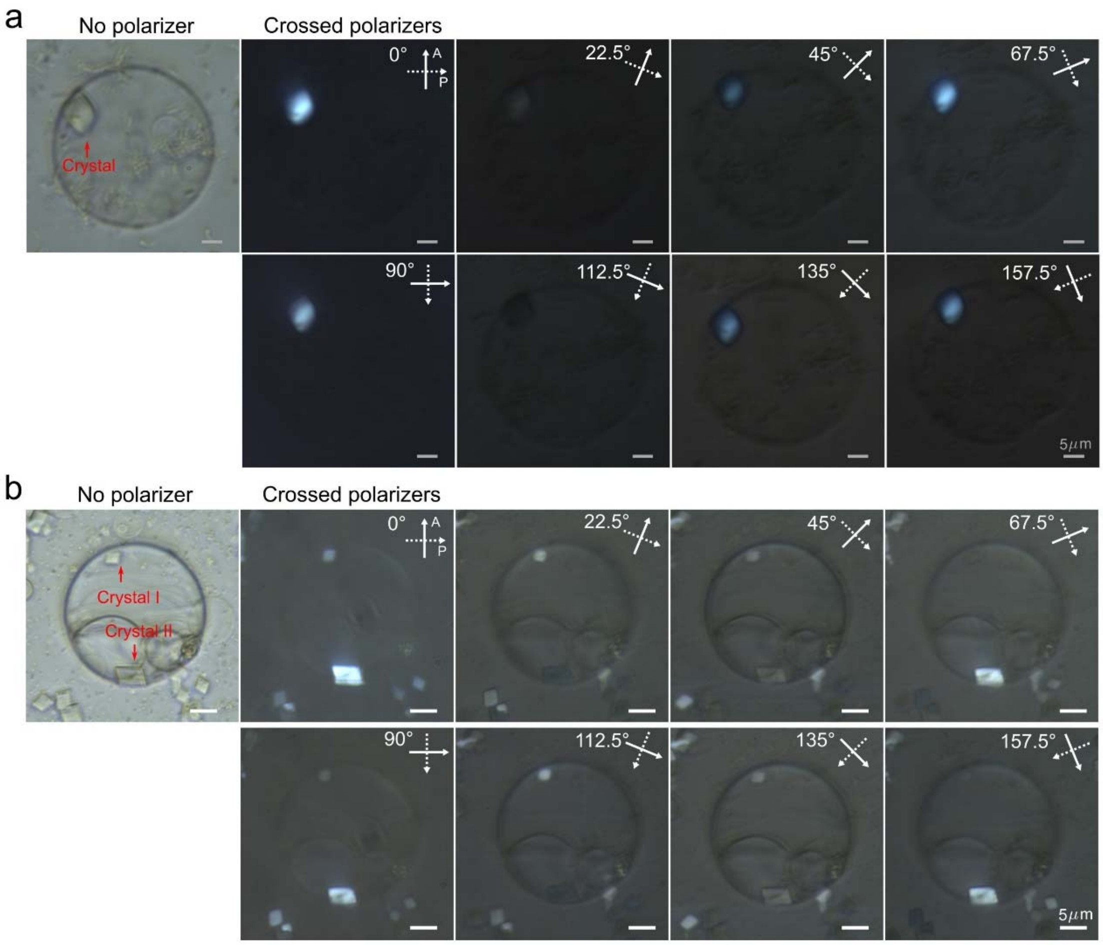

Figure S36. (**a**) A single DNA crystal and (**b**) two DNA crystals within DCMs. The DNA crystals appear bright and dark, when viewed through rotating crossed polarizers.

Supplementary Tables

Supplementary Table S1. Comparison of this work with previous DNA nanopore-based systems.

Aspect	Feature	This work	**Ref. 34** (Angew. Chem. Int. Ed. 2022, 61, e202210886.)	**Ref. 45** (Nat. Mater.2025, 24, 278-286.)
**System properties**	System composition	Fully artificial system integrating two distinct DNA-based pores within GUVs	Single DNA nanopore reconstituted into lipid membranes	Hybrid system combining natural OmpF protein pores, DNA-based pores, and GUVs
	Cyclic membrane remodeling	Yes	-	No
	Small pore (SP) size	~ 2 nm (DNA nanopore)	~ 2 nm (DNA nanopore)	~ 1.1 nm (OmpF protein)
	Large pore (LP) size	~ 15 nm	-	~ 15 nm
	Reversibility of SPs	Yes	Yes	No
	Re-opening of LPs	Yes	-	No
	Temporal control of pores	Light-responsive SPs and re-openable LPs enabling stepwise regulation	Light-induced on/off switching of SPs only	Not possible (OmpF permanently open)
**Mechanistic insights**	Experimental demonstration of inter-pore signaling	Clear demonstration via dynamic coordination between SPs and LPs through membrane re-shaping and recovery	-	Not possible (OmpF permanently open)
	Theoretical framework of LP formation	Yes	-	No
**Applications/ demonstrations**	Overall experimental summary	Double-necked synthetic cell microreactor enabling programmable, spatiotemporally regulated transport of reactants within cell-sized compartments	No application demonstration	Sequential reactant transport with limited controllability due to constitutively open OmpF
	Enzyme cascade	LPs regulate entry of large biomolecules, while SPs control influx of small solutes to initiate reactions	-	Stepwise reactant transport only
	Actin polymerization and bundling	Temporal control over cytoskeleton-relevant processes	-	-
	Confined synthesis of 3D DNA crystals	Controlled DNA crystallization exhibiting notable confinement effects	-	-
	Cell-free RNA transcription	Spatiotemporally controlled RNA transcription and fluorescence activation of Spinach RNA aptamers	-	-
**Comments**	Research focus	Versatile synthetic microreactor platform emulating and extending the functional complexity of natural cellular systems	Light-gated DNA nanopore for small-molecule transport regulation	Interface between reconfigurable DNA nanostructures and synthetic cells
	Perspective	Fully modular and robust artificial system	Functional versatility restricted by the single small-pore design	Protein extraction and stability constraints; incompatible with higher-order processes

Table S2. Sequences of the SP.

Name	Sequence (5'- 3')
S1	AGCGAACGTGGATTTTGTCCGACATCGGCAAGCTCCCTTTTTCGACTATT
S2-dock	CCGATGTCGGACTTCGTGCTCTTTTAGTCACGTTACACGATCTTCGCCTGCTGGGTTTTGGGAGCTTG
S3	CGAAGATCGTGTTTTTCCACAGTTGATTGCCCTTCACTTTTCCCAGCAGG
S4	AATCAACTGTGGTTTTTCTCACTGGTGATTAGAATGCTTTTGTGAAGGGC
S5-dock	TCACCAGTGAGATTCGCTGCGCGGTTTTTTTAAGTAATCACGTTTGTCGTACCAGGTGCATGGATTTTTGCATTCTAA
S6	CCTGGTACGACATTTTTCCACGTTCGCTAATAGTCGATTTTATCCATGCA
Lid-azo	GA/iAbz/GCA/iAbz/CGTTTTTTTTCGTGATTACTTATTTTTTTAACCGCGCAGCGTTTTTTTTCG/iAbz/TGA/iAbz/CT
8	GTTTGCCACTATCCGCGACCTGCTTCAACTAATGCAGATACATAACGCCAAAAGGAATTACG
S1-chol	*Chol-TEG*-AGCGAACGTGGATTTTGTCCGACATCGGCAAGCTCCCTTTTTCGACTATT
S2-dock-chol	*Chol-TEG*-CCGATGTCGGACTTCGTGCTCTTTTAGTCACGTTACACGATCTTCGCCTGCTGGGTTTTGGGAGCTTG
S3-chol	*Chol-TEG*-CGAAGATCGTGTTTTTCCACAGTTGATTGCCCTTCACTTTTCCCAGCAGG
S6-chol	*Chol-TEG*-CCTGGTACGACATTTTTCCACGTTCGCTAATAGTCGATTTTATCCATGCA
S4-Cy5	AATCAACTGTGGTTT/iCy5dT/TCTCACTGGTGATTAGAATGCTTTTGTGAAGGGC
lid-Cy3	GAGCACGTTTTTTTTCGTGATTACTTATTTTTTTAACCGCGCAGCG/iCy3dT/TTTTTTTTCGTGACT

Table S3. Sequences of the s-DR.

Name	Sequence (5' - 3')	5' End
1	AACGCCATCAAAAGTATAAGCAAATA	1[40]
2	GGCCTTCCTGTAGTGCTGCAAGGCGA	1[56]
3	ACATTAAATGTGAAATCATATGTACC	1[72]
4	CGTCGGATTCTCCGGCGATCGGTGCG	1[88]
5	GCGGATTGACCGTGTCTGGAGCAAAC	1[104]
6	ACGTTGGTGTAGAGGAAACCAGGCAA	1[120]
7	ACCGTGCATCTGCAGGGTAGCTATTT	1[136]
8	TTTAAATTGTAAATCAGAGCATAAAG	2[39]
9	AAACAGGAAGATTATAATTCGCGTCT	2[55]
10	CCGGTTGATAATCCTTTTGCGGGAGA	2[71]
11	AAACTAGCATGTCGCGAGTAACAACC	2[87]
12	AAGAGAATCGATGCTCATATATTTTA	2[103]
13	TCATTGCCTGAGAAATGGGATAGGTC	2[119]
14	TTGAGAGATCTACGGGTGAGAAAGGC	2[135]
15	CTAAATCGGTTGTCTACTAATAGTAG	3[40]
16	TGACCCTGTAATAAGAAAAGCCCCAA	3[56]
17	AGCCTTTATTTCACTGTTTAGCTATA	3[72]
18	AATTTTTAGAACCAACGGTAATCGTA	3[88]
19	AATGCAATGCCTGAACGAGTAGATTT	3[104]
20	TAAAGATTCAAAAAAAGGCTATCAGG	3[120]
21	CGGAGACAGTCAAGTACGGTGTCTGG	3[136]
22	TAGCATTAACATCTCAGAAGCAAAGC	4[39]
23	GGTGGCATCAATTACCAAAAACATTA	4[55]

Name	Sequence (5' - 3')	5' End
24	TTTTCATTTGGGGTCAAATATCGCGT	4[71]
25	AATGGTCAATAACACGCAAGGATAAA	4[87]
26	AGTTTGACCATTACTCCAACAGGTCA	4[103]
27	TCCCAATTCTGCGAGTAATGTGTAGG	4[119]
28	AAGTTTCATTCCAGTCATTTTTGCGG	4[135]
29	GGATTGCATCAAACCCTCAAATGCTT	5[40]
30	AGCCCGAAAGACTCGCGAGCTGAAAA	5[56]
31	TTTAATTCGAGCTAGACTGGATAGCG	5[72]
32	GACCGGAAGCAAAGATACATTTCGCA	5[88]
33	GGATTAGAGAGTAGAGAGGCTTTTGC	5[104]
34	CTTTTGATAAGAGTATAACAGTTGAT	5[120]
35	ATGGCTTAGAGCTGCAACACTATCAT	5[136]
36	TAAACAGTTCAGATTGGGCTTGAGAT	6[39]
37	ATTCATTGAATCCAAGATTAAGAGGA	6[55]
38	TCCAATACTGCGGCGATTTTAAGAAC	6[71]
39	TAGTAAAATGTTTTCAAAGCGAACCA	6[87]
40	AAAAGAAGTTTTGTACGTTAATAAAA	6[103]
41	AAACCAAAATAGCCCTTTAATTGCTC	6[119]
42	AACCCTCGTTTACATCAGTTGAGATT	6[135]
43	GGTTTAATTTCAAACCCAAATCAACG	7[40]
44	GAATTACCTTATGAATCGTCATAAAT	7[56]
45	TGGCTCATTATACAGGCTGGCTGACC	7[72]
46	GGGAAGAAAAATCCCAGAGGGGGTAA	7[88]
47	CGAACTAACGGAAAACTGACCAACTT	7[104]
48	GGTAGAAAGATTCCAGACGACGATAA	7[120]
49	TAGGAATACCACATCCATGTTACTTA	7[136]
50	TAACAAAGCTGCTGGGTAGCAACGGC	8[39]
51	CCGGATATTCATTCTTTAATCATTGT	8[55]
52	TTCATCAAGAGTAGTTTCCATTAAAC	8[71]
53	AGACCAGGCGCATCAGTCAGGACGTT	8[87]
54	TGAAAGAGGACAGAAACGAAAGAGGC	8[103]
55	CATAAGGGAACCGCAACATTATTACA	8[119]
56	GCCGGAACGAGGCATTATACCAAGCG	8[135]
57	TACAGAGGCTTTGTCGCTGAGGCTTG	9[40]
58	TTTTCATGAGGAAATCTTGACAAGAA	9[56]
59	GGGTAAAATACGTGTTGCGCCGACAA	9[72]
60	AGGCACCAACCTAATGAACGGTGTAC	9[88]
61	AAAAGAATACACTGGTTTATCAGCTT	9[104]
62	TTGACCCCCAGCGGCAGACGGTCAAT	9[120]
63	CGAAACAAAGTACCACGTTGAAAATC	9[136]
64	CAGGGAGTTAAAGCCCAATAGGAACC	10[39]
65	CCGATATATTCGGAGGACTAAAGACT	10[55]
66	TGACAACAACCATACAACGCCTGTAG	10[71]
67	CTTGATACCGATAAATGCCACTACGA	10[87]
68	GCTTTCGAGGTGAAAGTTTTGTCGTC	10[103]
69	CTTTAATTGTATCAAAACACTCATCT	10[119]
70	TCCAAAAAAAAGGTGCTAAACAACTT	10[135]
71	CATGTACCGTAACAGTACCGCCACCC	11[40]
72	ACCAGTACAAACTCGCCCACGCATAA	11[56]

Name	Sequence (5' - 3')	5' End
73	CATTCCACAGACAGGTTGATATAAGT	11[72]
74	GCGTAACGATCTAATTTCTTAAACAG	11[88]
75	TTTCCAGACGTTATTAGGATTAGCGG	11[104]
76	CTGTATGGGATTTCTCCAAAAGGAGC	11[120]
77	TCAACAGTTTCAGATTATTCTGAAAC	11[136]
78	TCAGAACCGCCACAGCATTGACAGGA	12[39]
79	ACTCAGGAGGTTTACTGAGTTTCGTC	12[55]
80	ATAGCCCGGAATAACAAATAAATCCT	12[71]
81	GTGCCGTCGAGAGGCCCTCATAGTTA	12[87]
82	GGTTTTGCTCAGTCGTTCCAGTAAGC	12[103]
83	CTCAAGAGAAGGAGTAAATGAATTTT	12[119]
84	ATGAAAGTATTAAATAAGTTTTAACG	12[135]
85	GGTTGAGGCAGGTGCCGCCACCCTCA	13[40]
86	TTGATATTCACAAGGTGTATCACCGT	13[56]
87	CATTAAAGCCAGACATAATCAAAATC	13[72]
88	TCTCTGAATTTACACCAGGCGGATAA	13[88]
89	GTCATACATGGCTTTTTCATCGGCAT	13[104]
90	GAGTGTACTGGTAGAGGCTGAGACTC	13[120]
91	GGGTCAGTGCCTTGTAATCAGTAGCG	13[136]
92	GAACCGCCACCCTTTTGTCACAATCA	14[39]
93	CGCCTCCCTCAGACAGACGATTGGCC	14[55]
94	ACCGGAACCAGAGGGGCGACATTCAA	14[71]
95	TTTGCCATCTTTTATGGAAAGCGCAG	14[87]
96	TTTCGGTCATAGCTCATTAAAGGTGA	14[103]
97	AGACTGTAGCGCGTTTGATGATACAG	14[119]
98	ACAGAATCAAGTTGCCAGCAAAATCA	14[135]
99	ATAGAAAATTCATGTTAGCAAACGTA	15[40]
100	GCCAAAGACAAAACCACCACCGGAAC	15[56]
101	CCGATTGAGGGAGAACGGAATACCCA	15[72]
102	TGACGGAAATTATCCCCTTATTAGCG	15[88]
103	ATTATCACCGTCAAAGCAGATAGCCG	15[104]
104	TTTGGGAATTAGATGCCTTTAGCGTC	15[120]
105	CCAGTAGCACCATGAAACAATGAAAT	15[136]
106	GAAAATACATACAGCCAGTTACAAAA	16[39]
107	TATTACGCAGTATATGGTTTACCAGC	16[55]
108	AAAGAACTGGCATGATTTTTTGTTTA	16[71]
109	GAAACGCAATAATGGAAGGTAAATAT	16[87]
110	AACAAAGTTACCAACATAAAAACAGG	16[103]
111	TTTTAAGAAAAGTCCGACTTGAGCCA	16[119]
112	AGCAATAGCTATCCAAAGTCAGAGGG	16[135]
113	TAAACAGCCATATAGTTGCTATTTTG	17[40]
114	CCAAATAAGAAACGATTAAGACTCCT	17[56]
115	ACGTCAAAAATGATTTTAGCGAACCT	17[72]
116	TACAGAGAGAATAGAAGGAAACCGAG	17[88]
117	GAAGCGCATTAGAGCAAGCAAATCAG	17[104]
118	TGAACACCCTGAATTACCGAAGCCCT	17[120]
119	TAATTGAGCGCTAAACAAGCAAGCCG	17[136]
120	CACCCAGCTACAAAATAAGAGAATAT	18[39]
121	TTAAATCAAGATTTATTTATCCCAAT	18[55]

Name	Sequence (5' - 3')	5' End
122	CCCGACTTGCGGGCGACAATAAACAA	18[71]
123	AGAACGCGAGGCGAAATAGCAGCCTT	18[87]
124	ATATAGAAGGCTTTAGATAAGTCCTG	18[103]
125	ACCGCGCCCAATACGGGAGAATTAAC	18[119]
126	TTTTTATTTTCATTAGAAACCAATCA	18[135]
127	AAAGTACCGACAAAACAGTAGGGCTT	19[40]
128	TCTGTCCAGACGAAGGTTTTGAAGCC	19[56]
129	CATGTTCAGCTAAGTTTAGTATCATA	19[72]
130	TGTTTATCAACAAATCCGGTATTCTA	19[88]
131	AACAAGAAAAATATAAGGCGTTAAAT	19[104]
132	ATTTACGAGCATGCGTAGGAATCATT	19[120]
133	ATAATCGGCTGTCTTTCATCTTCTGA	19[136]
134	AATTGAGAATCGCCTGTAAATCGTCG	20[39]
135	AAAGCCAACGCTCAAGGTAAAGTAAT	20[55]
136	TGCGTTATACAAACGATAGCTTAGAT	20[71]
137	CTAGAAAAAGCCTTGCAGAACGCGCC	20[87]
138	AAGAATAAACACCCATAGGTCTGAGA	20[103]
139	ACCGTGTGATAAAATATCCCATCCTA	20[119]
140	CCTAAATTTAATGACTATATGTAAAT	20[135]
141	CTATTAATTAATTTTTAACAATTTCA	21[40]
142	CTTGAAAACATAGTTCTTACCAGTAT	21[56]
143	TAAGACGCTGAGACTGAGCAAAAGAA	21[72]
144	AATTTATCAAAATGGAATCATAATTA	21[88]
145	GACTACCTTTTTATTGAATACCAAGT	21[104]
146	GTTGGGTTATATAGTTTGAAATACCG	21[120]
147	GCTGATGCAAATCGTAACAGTACCTT	21[136]
148	TTTGAATTACCTTTGGCCCTGAGAGA	22[39]
149	AAAATTAATTACATTCCCTTAGAATC	22[55]
150	GATGATGAAACAAGAAAATCCTGTTT	22[71]
151	TCATTTCAATTACAGAGTCAATAGTG	22[87]
152	TACAAAATCGCGCAAAGAATAGCCCG	22[103]
153	TCGCCTGATTGCTACCTCCGGCTTAG	22[119]
154	TTACATCGGGAGACACTATTAAAGAA	22[135]

Table S4. Staples with extension for hybridization with the TEG-cholesterol modified DNA strands (cholesterol sites) on the DNA rafts.

Name	Sequence (5'—3')	5' End
Chol-ST	*Chol-TEG*-TTTTTAACCAGACCACCCATAGCAT	
4[39]-C	TAGCATTAACATCTCAGAAGCAAAGCTTTTATGCTATGGGTGGTCTGGTT	4[39]
4[71]-C	TTTTCATTTGGGGTCAAATATCGCGTTTTTATGCTATGGGTGGTCTGGTT	4[71]
4[103]-C	AGTTTGACCATTACTCCAACAGGTCATTTTATGCTATGGGTGGTCTGGTT	4[103]
4[135]-C	AAGTTTCATTCCAGTCATTTTTGCGGTTTTATGCTATGGGTGGTCTGGTT	4[135]
12[39]-C	TCAGAACCGCCACAGCATTGACAGGATTTTATGCTATGGGTGGTCTGGTT	12[39]
12[71]-C	ATAGCCCGGAATAACAAATAAATCCTTTTTATGCTATGGGTGGTCTGGTT	12[71]
12[103]-C	GGTTTTGCTCAGTCGTTCCAGTAAGCTTTTATGCTATGGGTGGTCTGGTT	12[103]
12[135]-C	ATGAAAGTATTAAATAAGTTTTAACGTTTTATGCTATGGGTGGTCTGGTT	12[135]
16[39]-C	GAAAATACATACAGCCAGTTACAAAATTTTATGCTATGGGTGGTCTGGTT	16[39]
16[71]-C	AAAGAACTGGCATGATTTTTTGTTTATTTTATGCTATGGGTGGTCTGGTT	16[71]
16[103]-C	AACAAAGTTACCAACATAAAAACAGGTTTTATGCTATGGGTGGTCTGGTT	16[103]
16[135]-C	AGCAATAGCTATCCAAAGTCAGAGGGTTTTATGCTATGGGTGGTCTGGTT	16[135

Table S5. Sequences of unlocking DNA strands to reconfigure s-DRs to e-DRs.

Name	Sequence (5'- 3'), Unlocking DNA strands	5' End
1	TAATACTAGATTAAAATTCGCATTAAATTTTTGTTAAATCAGCTCATTTTGTAAAACGACGG	2[23]
2	AATTCTAGCAATTAATGCCGGAGCAGTTTGAGGGGACGACGACAGTATCGGCCTCAGGAAGA	2[151]
3	ATCCTTATATAGGCAAGGCAAAGAATTAGCAAAATTAAGCAATAAAGCCCGTTAATATTTG	4[23]
4	TTAGCTATTAATATGCAACTAAAATCACCATCAATATGATATTCAACCGTTCTAGCTGATAA	4[151]
5	TTGACGATACCATAAATCAAAAATCAGGTCTTTACCCTGACTATTATAGCAATAAATCATAC	6[23]
6	GACAATTTGTAGGCATAGTAAGATAATTGCTGAATATAATGCTGTAGCTCAACATGTTTTAA	6[151]
7	GCTCCAAAAGAGGCTTGCCCTGACGAGAAACACCAGAACGAGTAGTAAAAAACGAGAATGAC	8[23]
8	GTTTGCCACTATCCGCGACCTGCTTCAACTAATGCAGATACATAACGCCAAAAGGAATTACG	8[151]
9	ACCACATGCGGATCGTCACCCTCAGCAGCGAAAGACAGCATCGGAACGACATTCAGTGAATA	10[23]
10	TTCTCTGTAGATAATAATTTTTTAACGGAGATTTGTATCATCGCCTGATAAATTGTGTCGAA	10[151]
11	AGTAGCATCCACCCTCAGAGCCACCACCCTCATTTTCAGGGATAGCAAGGCCGCTTTTGCGG	12[23]
12	TATGGACCCGTATTTCGGAACCTCGGAGTGAGAATAGAAAGGAACAACTAAAGGAATTGCGA	12[151]
13	CAACTGTAGACTCAGAGCCGCCACCAGAACCACCACCAGAGCCGCCGCCCCTCAGAACCGCC	14[23]
14	GTCTAGGACGCGATAGCAGCACCGAGTAACAGTGCCCGTATAAACAGTTAATGCCCCCTGCC	14[151]
15	TGAAGTGGACATATAAAAGAAACGCAAAGACACCACGGAATAAGTTTATCAGAGCCACCACC	16[23]
16	GAGGAACCTAATAATAAGAGCAATACCATTAGCAAGGCCGGAAACGTCACCAATGAAACCAT	16[151]
17	CCAGTCAATACTTACCAACGCTAACGAGCGTCTTTCCAGAGCCTAATTTTAAAGGTGGCAAC	18[23]
18	CACGATAGGGCGCACTCATCGAGATATCAGAGAGATAACCCACAAGAATTGAGTTAAGCCCA	18[151]
19	TCCAAGCGATGCCAACATGTAATTTAGGCAGAGGCATTTTCGAGCCAGTTTTTATCCTGAAT	20[23]
20	CCTTTAAGCAATATTTTAGTTAATTTCCTTATCATTCCAAGAACGGGTATTAAACCAAGTAC	20[151]
21	AAAGAGCTCGAGTACATAAATCAATATATGTGAGTGAATAACCTTGCTTCATATTTAACAAC	22[23]
22	CCTTTAAGCAAGATGAATATACACAATCGCAAGACAAAGAACGCGAGAAAACTTTTTCAAAT	22[151]

Table S6. Releasing DNA strands with toehold triggers and square - lock DNA strands (these two types of DNA strands are called together as Locking DNA strands) to reconfigure the e-DRs back to R-s-DRs.

Name	Sequence (5'- 3'), Locking DNA strands	5' End
	**Square - lock DNA strands**	
1	TTAAGTTGGGTAACGCCAGGGTTTTCCCAGTCACGACGTTTTAACCAATAGG	0[55]
2	GGCCTCTTCGCTATTACGCCAGCTGGCGAAAGGGGGATGCCAGCTTTCATCA	0[87]
3	AGCGCCATTCGCCATTCAGGCTGCGCAACTGTTGGGAAGGTGGGAACAAACG	0[119]
4	TCGCACTCCAGCCAGCTTTCCGGCACCGCTTCTGGTGCCTGGGCGCATCGTA	0[151]
5	ACCAGTGAGACGGGCAACAGCTGATTGCCCTTCACCGCCTTTTAATGGAAAC	23[8]
6	GTTGCAGCAAGCGGTCCACGCTGGTTTGCCCCAGCAGGCACATCAAGAAAAC	23[40]
7	GATGGTGGTTCCGAAATCGGCAAAATCCCTTATAAATCAAGAGGCGAATTAT	23[72]
8	AGATAGGGTTGAGTGTTGTTCCAGTTTGGAACAAGAGTCAACAATAACGGAT	23[104]
9	CGTGGACTCCAACGTCAAAGGGCGAAAAACCGTCTATCACAGGTTTAACGTC	23[136]
	**Releasing DNA strands with toehold triggers**	
23	CCGTCGTTTTACAAAATGAGCTGATTTAACAAAAATTTAATGCGAATTTTAATCTAGTATTA	
24	TCTTCCTGAGGCCGATACTGTCGTCGTCCCCTCAAACTGCTCCGGCATTAATTGCTAGAATT	
25	CAAAATATTAACGGGCTTTATTGCTTAATTTTGCTAATTCTTTGCCTTGCCTATATAAGGAT	
26	TTATCAGCTAGAACGGTTGAATATCATATTGATGGTGATTTTAGTTGCATATTAATAGCTAA	
27	GTATGATTTATTGCTATAATAGTCAGGGTAAAGACCTGATTTTTGATTTATGGTATCGTCAA	
28	TTAAAACATGTTGAGCTACAGCATTATATTCAGCAATTATCTTACTATGCCTACAAATTGTC	
29	GTCATTCTCGTTTTTTACTACTCGTTCTGGTGTTTCTCGTCAGGGCAAGCCTCTTTTGGAGC	
30	CGTAATTCCTTTTGGCGTTATGTATCTGCATTAGTTGAAGCAGGTCGCGGATAGTGGCAAAC	
31	TATTCACTGAATGTCGTTCCGATGCTGTCTTTCGCTGCTGAGGGTGACGATCCGCATGTGGT	
32	TTCGACACAATTTATCAGGCGATGATACAAATCTCCGTTAAAAAATTATTATCTACAGAGAA	

Name	Sequence (5’- 3’), Locking DNA strands	5’ End
33	CCGCAAAAGCGGCCTTGCTATCCCTGAAAATGAGGGTGGTGGCTCTGAGGGTGGATGCTACT	
34	TCGCAATTCCTTTAGTTGTTCCTTTCTATTCTCACTCCGAGGTTCCGAAATACGGGTCCATA	
35	GGCGGTTCTGAGGGGCGGCGGCTCTGGTGGTGGTTCTGGTGGCGGCTCTGAGTCTACAGTTG	
36	GGCAGGGGGCATTAACTGTTTATACGGGCACTGTTACTCGGTGCTGCTATCGCGTCCTAGAC	
37	GGTGGTGGCTCTGATAAACTTATTCCGTGGTGTCTTTGCGTTTCTTTTATATGTCCACTTCA	
38	ATGGTTTCATTGGTGACGTTTCCGGCCTTGCTAATGGTATTGCTCTTATTATTAGGTTCCTC	
39	GTTGCCACCTTTAAAATTAGGCTCTGGAAAGACGCTCGTTAGCGTTGGTAAGTATTGACTGG	
40	TGGGCTTAACTCAATTCTTGTGGGTTATCTCTCTGATATCTCGATGAGTGCGCCCTATCGTG	
41	ATTCAGGATAAAAACTGGCTCGAAAATGCCTCTGCCTAAATTACATGTTGGCATCGCTTGGA	
42	GTACTTGGTTTAATACCCGTTCTTGGAATGATAAGGAAATTAACTAAAATATTGCTTAAAGG	
43	GTTGTTAAATATGAAGCAAGGTTATTCACTCACATATATTGATTTATGTACTCGAGCTCTTT	
44	ATTTGAAAAAGTTTTCTCGCGTTCTTTGTCTTGCGATTGTGTATATTCATCTTGCTTAAAGG	

Table S7. Staples with extension for hybridization with the Atto488 modified DNA strands and FRET pair modified staple DNA strands on the DNA rafts.

Name	Sequence (5’-3’)	5’ End
Atto488-ST	Atto488-GGGTTTGGTGTTTTTT	
Atto-1	AAAAAACACCAAACCCTTTTTTTAAATTGTAAATCAGAGCATAAAG	2[39]
Atto-2	AAAAAACACCAAACCCTCCGGTTGATAATCCTTTTGCGGGAGA	2[71]
Atto-3	AAAAAACACCAAACCCTTTTAAGAGAATCGATGCTCATATATTTTA	2[103]]
Atto-4	AAAAAACACCAAACCCTTTTTTTGAGAGATCTACGGGTGAGAAAGGC	2[135]
Atto-5	AAAAAACACCAAACCCTTTTAATTGAGAATCGCCTGTAAATCGTCG	20[39]
Atto-6	AAAAAACACCAAACCCTTTTTGCGTTATACAAACGATAGCTTAGAT	20[71]
Atto-7	AAAAAACACCAAACCCTTTTAAGAATAAACACCCATAGGTCTGAGA	20[103]
Atto-8	AAAAAACACCAAACCCTTTTCCTAAATTTAATGACTATATGTAAAT	20[135]
FRET-Cy3	Cy3- AGCATGTCGCGAGTAACAACC	2[87]
FRET-Cy5	Cy5-TTTCCGGTTGATAATCCTTTTGCGGGAGA	2[71]

Table S8. Sequences of the DNA template for Spinach RNA transcription and DNA motif for DNA crystal assembly.

	Name	Sequence (5’- 3’)
DNA template	T1	Cy5- TTTTTCGCTAATACGACTCACTATA
	T2	GGAGCTCACACTCTACTCAACAGTAGCGAACTACTGGACCCGT CCTTCACCCTATAGTGAGTCGTATTAGCGAGTATAGGG
DNA motif	L	CGCACCGCGCACCGCGCACCG
	S	p-TCTTTTGTGGTTTT
	M	p-GAAAAACCTGCGCGGACAAAA
	L-Cy5	Cy5-CGCACCGCGCACCGCGCACCG

Note: p means the 5′-phosphorylation.

References

1 Dixit, M. & Lazaridis, T. Free energy of hydrophilic and hydrophobic pores in lipid bilayers by free energy perturbation of a restraint. *J. Chem. Phys.* **153**, 054101 (2020).

2 Shin, H., Bowick, M. J. & Xing, X. Topological Defects in Spherical Nematics. *Phys. Rev. Lett.* **101**, 037802 (2008).
